# Internet-Based Interventions for Carers of Individuals With Psychiatric Disorders, Neurological Disorders, or Brain Injuries: Systematic Review

**DOI:** 10.2196/10876

**Published:** 2019-07-09

**Authors:** Lucy Spencer, Rachel Potterton, Karina Allen, Peter Musiat, Ulrike Schmidt

**Affiliations:** 1 Section of Eating Disorders Institute of Psychiatry, Psychology, and Neuroscience King’s College London London United Kingdom; 2 The Eating Disorders Service Maudsley Hospital South London & Maudsley National Health Service Foundation Trust London United Kingdom

**Keywords:** internet, carers, mental health, technology, review

## Abstract

**Background:**

Nonprofessional carers who provide support to an individual with a psychiatric or neurological disorder will often themselves experience symptoms of stress, anxiety, or low mood, and they perceive that they receive little support. Internet-based interventions have previously been found to be effective in the prevention and treatment of a range of mental health difficulties in carers.

**Objective:**

This review seeks to establish the status of internet-based interventions for informal (nonprofessional) carers of people with psychiatric or neurological disorders by investigating (1) the number and quality of studies evaluating the efficacy or effectiveness of internet-based carer interventions and (2) the impact that such interventions have on carer mental health, as well as (3) how internet-based interventions compare with other intervention types (eg, face-to-face treatment).

**Methods:**

A systematic literature search was conducted in January 2019 using the EMBASE (1974-present), Ovid MEDLINE (1946-present), PsychARTICLES, PsychINFO (1806-present), and Global Health (1973-present) databases, via the Ovid Technologies database. Search terms included carer, caregiver, online, technology, internet-based, internet, interactive, intervention, and evaluation. Studies selected for inclusion in this review met the following predetermined criteria: (1) delivering an intervention aimed primarily at informal carers, (2) carers supporting individuals with psychiatric disorders, stroke, dementia, or brain injury, (3) the intervention delivered to the carers was primarily internet based, (4) the study reported a pre- and postquantitative measure of carer depression, anxiety, stress, burden, or quality of life, (5) appeared in a peer-reviewed journal, and (6) was accessible in English.

**Results:**

A total of 46 studies were identified for inclusion through the detailed search strategy. The search was conducted, and data were extracted independently by 2 researchers. The majority of studies reported that 1 or more measures relating to carer mental health improved following receipt of a relevant intervention, with interventions for carers of people with traumatic brain injury showing a consistent link with improved outcomes.

**Conclusions:**

Studies investigating internet-based interventions for carers of individuals with diverse psychiatric or neurological difficulties show some evidence in support of the effectiveness of these interventions. In addition, such interventions are acceptable to carers. Available evidence is of varying quality, and more high-quality trials are needed. Further research should also establish how specific intervention components, such as structure or interactivity, contribute to their overall efficacy with regard to carer mental health.

## Introduction

### Background

Nonprofessional (or informal) carers are individuals who provide free-of-charge care for another person (usually a family member or friend), who would find it difficult to cope without the carer’s support. Informal carers play a crucial role in providing both practical and emotional care for individuals with a wide range of difficulties, including physical and mental health difficulties, disabilities, or addictions. It is estimated that during 2015, 6.8 million people in the United Kingdom provided unpaid care to a close other, a 16.5% increase from 2001, reflecting an economic value of £132 billion of informal care per year, almost double the value in 2001 [1]. In addition, the support of informal carers may reduce and delay hospital admissions [2], thus further reducing the burden on health care systems.

It has been widely documented that caring for a loved one with a long-term illness can have a multitude of effects on the informal carer, including increased levels of perceived burden [3], feelings of entrapment, shame, guilt [4], and higher rates of physical symptoms, such as fatigue, headaches, and weight loss [5]. It is important to recognize that disorder- or patient-related factors can profoundly impact carers’ experience of caregiving. Such factors may include the nature and severity of different symptoms, societal reactions, and certain pathologies, which may vary widely both within and between disorders. For example, when comparing carers of people with schizophrenia and carers of people with long-term physical disorders, levels of subjective burden were found to be higher in carers of people with schizophrenia and brain diseases than in other groups [6]. Furthermore, levels of social support available to carers of individuals with schizophrenia were found to be lower than for carers of individuals with physical disorders.

In addition to the differential impact that various diagnoses and symptoms can have on carer difficulties, it is also important to consider how individual differences in perceptions of burden affect the experience of caring. Caregiver Identity Theory [7] posits that the main source of carer distress is identity discrepancy—the disparity between the activities they are required to carry out as a carer and their own views of self (or *identity standard*). This may explain why there is a wide variation in perceived burden or distress in carers who, on the face of it, are required to carry out similar caring responsibilities (eg, carers of people with dementia), and it may be a helpful dimension to explore when considering possible interventions or support plans. A range of carer-focused face-to-face interventions, often delivered in group formats, have been found to reduce psychological distress and improve the quality of life of individuals caring for people with severe mental health difficulties [8]. However, some carers may find it difficult to attend regular appointments because of time constraints, or they may have concerns regarding privacy or stigma [9]. Internet-based interventions have been found to be effective in prevention and treatment of a range of psychiatric disorders, including depression [10], anxiety [11], posttraumatic stress disorder [12], and eating disorders [13]. As carers often experience elevated levels of depression and stress, as well as reduced general well-being [14], internet interventions to improve carers’ own mental health should be considered as a potentially viable option. Previous reviews of the impact of internet-based interventions on carer distress have focused on carers of people with a broad range of mental and physical disorders (including dementia, cancer, mental health difficulties, and hip fractures), and they have reported positive or mixed findings [15,16]. In contrast, this review focuses specifically on carers of people with psychiatric disorders, neurological disorders (dementia, stroke), or brain injury. We decided to restrict our inclusion criteria to carers of individuals with these disorders specifically, as the burden of caring for someone whose primary difficulty relates to his or her cognitive abilities or mental health may be very different from that of caring for someone whose condition primarily impacts on his or her physical health (eg, cancer). Some studies focus on whether internet-based interventions can help increase carers’ knowledge of their loved one’s disorder [17] or teach them relevant skills to manage or change their loved one’s behavior—for example, children with attention-deficit hyperactivity disorder (ADHD) [18].

### Objectives

Although improving carer knowledge and skills is important, this review focuses on whether internet-based interventions can improve carer mental health, and this review has therefore only included studies that measure aspects of this, such as depression, anxiety, stress, burden, or perceived quality of life. Specifically, this review seeks to establish the status of internet-based interventions for informal carers of people with psychiatric or neurological disorders by investigating (1) the number and quality of studies evaluating the efficacy or effectiveness of internet-based carer interventions and (2) the impact that such interventions have on carer mental health, (3) as well as how internet-based interventions compare with other intervention types (eg, face-to-face treatment).

## Methods

### Eligibility Criteria

The papers selected for inclusion in this review met the following predetermined criteria: (1) delivering an intervention aimed primarily at informal (nonprofessional) carers of (2) children or adults with psychiatric disorders, stroke, dementia, or brain injury; (3) the intervention delivered to the carers was primarily internet based, (4) the study reported a pre- and postquantitative measure of carer depression, anxiety, stress, burden, or quality of life, (5) appeared in a peer-reviewed journal, and (6) was accessible in English. Studies were excluded if the intervention was aimed exclusively at the patient rather than the carer. Papers exclusively reporting other measures of intervention efficacy (eg, increase in carer knowledge) were also excluded from this review.

### Information Sources

A systematic literature search was conducted in January 2019 using the EMBASE (1974-present), Ovid MEDLINE (1946-present), PsychARTICLES, PsychINFO (1806-present), and Global Health (1973-present) databases, via the Ovid Technologies database. Searches of reference lists of articles listed in this review, as well as relevant review papers, were also conducted. A total of 5 papers identified in the literature search, which could not be obtained, were requested from their authors via ResearchGate. Of these, 2 authors responded by sending the full text of their study to be assessed for eligibility. The search was limited to papers that could be accessed in English.

### Search Strategy

Search terms were the following; (carer OR caregiver OR care-giver OR carers) AND (online OR on-line OR technology OR internet-based OR interactive OR internet) AND (intervention OR evaluation). This search strategy returned 46 studies that met each of the inclusion criteria detailed above.

### Data Collection Process

A data extraction sheet (adapted from the Cochrane Consumers and Communication Review Group’s data extraction template) was developed and pilot tested on 5 randomly selected studies to be included in the review and refined accordingly following the pilot testing. Data were extracted from the included studies by 1 study author (LS) and checked by a second author (RP). Disagreements between reviewers were resolved by consensus. No authors were contacted for further information.

### Data Items

For each study within the review, we extracted participant characteristics (number per study arm, average age, and gender), details of the internet-based intervention (including intervention name, content, average number of sessions, and duration), and details of the control group where applicable. Regarding study findings, we extracted data regarding the statistical significance of quantitative findings of carer psychological health and the time points at which outcome data were collected. In addition, we identified the primary outcome(s) where this was specified and extra data regarding any qualitative findings with particular relevance to carer outcomes and mental health.

### Rating Evidence of Intervention Effectiveness

Each of the studies included in the review was rated on the effectiveness of the intervention employed, in terms of the extent to which the intervention had a statistically significant impact on outcomes relating to carer depression, anxiety, stress, burden, or quality of life. Studies were given 1 of 3 ratings:

- Intervention shows *clear association* with positive outcomes relating to carer depression, anxiety, stress, burden, or quality of life (half or more outcome measures show statistically significant and positive impact of intervention). *Effectiveness of intervention score=3.*
- Intervention shows *some association* with positive outcomes relating to carer depression, anxiety, stress, burden, or quality of life (fewer than half, but at least one outcome measure show(s) statistically significant and positive impact of intervention). *Effectiveness of intervention score=2.*
- Intervention shows *very little or no association* with positive outcomes relating to carer distress, anxiety, stress, burden, or quality of life (no outcomes showing a statistically significant and positive impact of intervention). *Effectiveness of intervention score=1.*

### Risk of Bias in Individual Studies

Risk of bias within individual studies was rated independently by 2 researchers (LS and RP). Studies were assessed using a scale developed previously to evaluate risk of bias and study quality in a review of interventions for individuals with anorexia nervosa (AN) [19]. A table displaying the risk of bias of each individual study can be found in Multimedia Appendix 1. Randomized controlled trials (RCTs) were given a rating out of 20 (the total number of items). RCTs receiving a score of 14 or over were deemed to be *high quality*, RCTs receiving a score of above 8 and below 14 were deemed to be of *moderate quality*, and RCTs studies receiving a score of 8 or below were deemed to be *low quality*. Studies that did not employ an RCT design were rated on the 13 items of the scale relevant to non-RCTs. Of these non-RCT studies, those receiving a score of 10 or over were deemed to be *high quality*, those receiving a score of above 6 and below 10 were deemed to be of *moderate quality*, and those receiving a score of 6 or below were deemed to be *low quality*. Disagreements were resolved by discussion. Of the 46 studies identified for inclusion in the review, 16 RCTs and 0 non-RCTs were rated as high quality, 11 RCTs and 6 non-RCTs were rated as moderate quality, and 2 RCTs and 11 non-RCTs were rated as low quality.

### Study Selection

Eligibility assessment for each study to be included in the systematic review was performed independently by 2 reviewers (LS and RP), using the eligibility criteria detailed above. Disagreements between reviewers were resolved by consensus. A search of EMBASE, Ovid MEDLINE, PsychARTICLES, PsychINFO, and Global Health returned a total of 3458 studies. A total of 5 additional citations were identified through searching the reference lists of relevant papers. After a preliminary screening, the full text of 238 articles were examined in detail, of which 192 were excluded. Reasons for the exclusion of the studies examined in detail can be found in Table 1.

**Figure 1:**
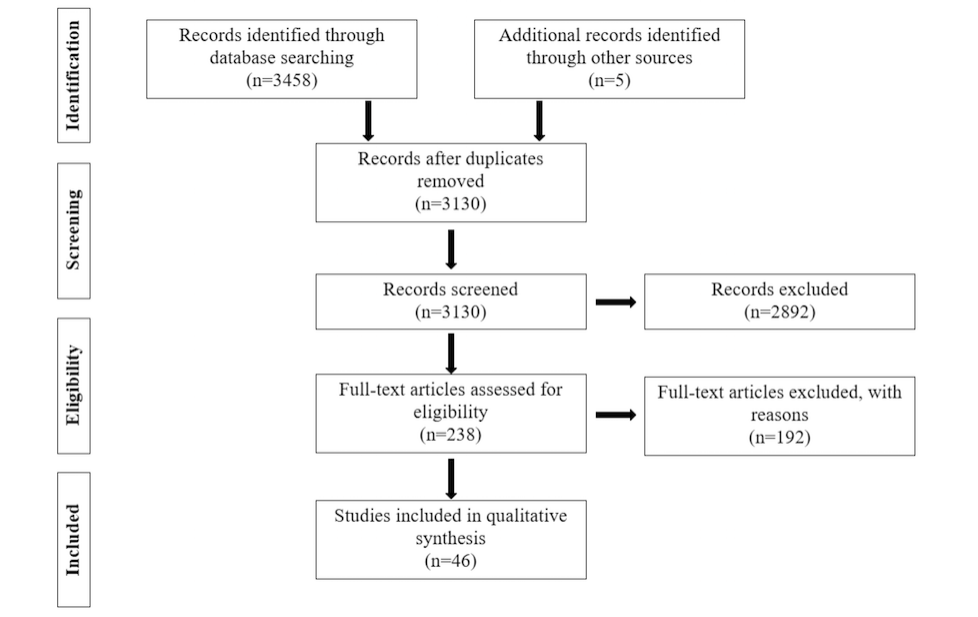
Flowchart of study selection for the review.

Agreement between the 2 reviewers during the selection of abstracts, as measured by Cohen kappa, was 0.860, and agreement during selection of full texts for inclusion was 0.892, both of which are regarded as excellent. A total of 46 studies met inclusion criteria and were included in the review. A flow diagram detailing the selection of studies for inclusion can be found in Figure 1.

**Table 1:** Reasons for exclusion of studies examined in detail (n=192).

Reason for excluding study	Studies excluded, n (%)
Review article	27 (14.1)
Does not report an intervention	10 (5.2)
Qualitative data only	16 (8.3)
Carer recipient not suffering from mental health, dementia, stroke, or brain injury	22 (11.5)
Unable to access	12 (6.25)
Not available in English	4 (2.1)
Not an article in a peer-reviewed journal	28 (14.6)
Does not report pre/post measure of burden, stress, depression, anxiety, or quality of life	25 (13.0)
Study protocol	11 (5.7)
Does not report an internet-based intervention	29 (15.1)
Intervention aimed at sufferer rather than carer	6 (3.1)
Professional caregivers	2 (1.0)

## Results

### Study Quality

Of the 46 studies identified for inclusion in the review, 16 RCTs and 0 non-RCTs were rated as high quality, 11 RCTs and 6 non-RCTs were rated as moderate quality, and 2 RCTs and 11 non-RCTs were rated as low quality.

### Psychiatric Disorders

Studies regarding carers of individuals with psychiatric disorders are summarized in Multimedia Appendix 2. A total of 12 such studies were identified [18,20-30]. For the purpose of this review, studies are grouped and presented below by specific disorder (AN, Schizophrenia or Schizoaffective disorder, ADHD, and studies describing mixed mental health difficulties).

#### Anorexia Nervosa

A total of 2 RCTs explored the efficacy of a sequential, 8-modular (with participants completing approximately 1 module per week) internet-based intervention (Overcoming Anorexia Online; OAO), based upon a systemic, cognitive behavioral therapy (CBT) framework, in supporting carers of adults with AN. In a study rated to be of high quality, in a comparison of carers receiving OAO to those receiving support as usual from the charity B-eat, OAO was found to reduce levels of carer anxiety and depression to a greater extent than support as usual [20]. In a later study, rated to be of moderate quality, the OAO intervention with clinician guidance was compared with OAO without additional guidance [21]. No significant improvements were found in either group over time, regarding carer anxiety, depression, or stress. Improvements over time were observed across other measures (including negative experiences of caregiving and intrusiveness), although some of these improvements were observed in the group receiving clinician guidance, and some were observed in the group that did not receive guidance.

#### Depression

In a high-quality RCT, participation in a self-management intervention, “E-care for Caregivers”, for a period of 6 weeks [22] was not found to be associated with a decrease in carer distress. However, the intervention was rated as user friendly by participants, indicating that the further development and implementation of internet-based interventions may be acceptable to carers of individuals with depression.

#### Schizophrenia and Schizoaffective Disorder

The efficacy of a telehealth intervention (“The Schizophrenia Guide”) of 3 months duration, for individuals with schizophrenia (or schizoaffective disorder) and their carers, was compared within an RCT with care as usual, in a study considered to be of moderate quality. No between-group differences were found with regard to levels of perceived carer stress. However, individuals with schizophrenia allocated to the intervention group were found to display reduced stress levels [23]. Another study investigated the efficacy of a internet-based, multifamily intervention [24], compared with support as usual in a quasi-experimental trial, with the interventions delivered over a period of 12 months. This study also did not find a significant difference in levels of carer distress between intervention and control groups. However, family relationship stress improved over time in the intervention group, and the majority of users expressed high levels of satisfaction with the internet-based intervention.

#### Attention-Deficit Hyperactivity Disorder

A small case series (n=8) investigating the impact of an 8-session, psychoeducational parenting program, delivered via videoconferencing, found improvements in both parent distress and child behavior over time [18]. In a recent study, 6 sessions of behavior training delivered over an intervention period of 25 weeks via videoconferencing technology was compared with the same intervention content, delivered in person to participants [25]. Carers allocated to the videoconferencing group did not report improvements in their own mental health, whereas those in the in-person group did. Families in both conditions reported similar levels of improvement in their child’s level of functioning and comparable satisfaction.

#### Mixed Mental Health Difficulties

Stjernswärd and Hansson [26], in a high quality RCT, compared a 10-week internet-based mindfulness program with a wait-list control, in adult carers supporting people with a wide range of diagnoses (including depression, anxiety disorders, psychosis, and autism spectrum disorders), and they found that, in addition to an improvement in mindfulness, those in the intervention group also reported a reduction in perceived stress and some aspects of quality of life and carer burden. In addition, the same authors reported results of a prepost comparison, in which participants completed an 8-week internet-based mindfulness program [27]. Similarly to the results of their RCT, improvements were found in carer quality of life and burden, perceived stress, and mindfulness. These improvements were largely maintained at 3-month follow-up. In their most recent study, these authors reported the effectiveness of a similar internet-based mindfulness program on a large number (n=398) of carers of people with mental or somatic illnesses [28]. Improvements in carer stress were again found in the intervention group at 8 weeks, maintained at follow-up. Within the experimental group, burden was found to decrease from pre to follow-up on both the objective and subjective subscales. A recent prepost comparison study [29] targeting carers of adolescents with mental health problems found that a 3-month intervention powered by a Moderated Online Social Therapy software platform was acceptable and safe for use by participants, and it found that after engaging with the program, participants showed a significant reduction on a measure of stress, although other measures relating to carer mental well-being (depression, anxiety, and psychological well-being) had not changed significantly by the end of the intervention. Finally, a recent RCT (considered to be of high quality) specifically focused on the needs of young carers (aged 16-25 years) of individuals with mental illness. This study compared the effectiveness of a internet-based intervention with “folder support” (participants in this condition were provided with a folder, containing information on available support services). Results regarding the efficacy of the internet-based intervention were mixed [30]. No between-group differences were observed with regard to carer stress, and although both groups displayed an increase in well-being, only the folder group displayed improvements in carer self-efficacy and quality of life.

### Stroke

A total of 4 studies (summarized in Multimedia Appendix 3) were identified, which tested internet-based interventions for carers of stroke survivors [31-34]. A total of 2 of these were RCTs, and they were rated as being of moderate and high quality, respectively [32,33], with the other 2 studies [31,34], comprising prepost comparisons, rated to be of low quality. Ranging in duration from 4 weeks [31] to 12 months [32,34], each of the 4 interventions provided relevant informative resources, in addition to contact with fellow carers and professionals through a range of channels, including email, message boards, internet-based chats, telephone, and videophone. With regard to carer psychological functioning, 2 of the 4 studies found no significant change in measures of depression, life satisfaction [32], burden, or mental health [34] in participants receiving the respective internet-based interventions. However, Smith et al [33] found a significant improvement in reported depression (including a clinically meaningful change) in the intervention group over time, whereas Graf et al [31] reported a decrease in both depressive symptoms and burden, regardless of total number of years spent caring. There appeared to be a relationship between intervention duration and carer outcomes—the 2 studies finding significant improvements over time [31,33] lasted 4 and 11 weeks, respectively, whereas the 2 of longer duration (12 months) [32,34] did not find any significant change in carer outcomes.

### Dementia

We identified a total of 22 studies documenting internet interventions for carers of people with dementia. A total of 12 of these included a comparison group (Multimedia Appendix 4; [17,35-45]), and 10 studies were conducted without a control group (Multimedia Appendix 5; [34,46-54]). Of the studies featuring a control group, 5 were rated as high quality, 4 were rated as moderate quality, and 3 were rated as low quality. Of the studies without a control group, 4 were rated as moderate quality, and 6 were rated as low quality. Intervention duration varied from 30 days [35] to 12 months [34,36,37].

Again, these studies reported mixed findings with regard to the impact of the interventions on measures of carer mental health, with 8 of the interventions showing a clear positive impact on carer outcomes (effectiveness score of 3), 5 of the interventions showing some positive impact on carer outcomes (effectiveness score of 2), and 9 found to have little or no positive impact on carer outcomes (effectiveness score of 1). Interventions reporting clear positive findings tended to be those of shorter duration, with 4 of the 8 lasting 9 weeks or less [35,46-48], in comparison to interventions reporting little or no positive impact, of which only 2 of the 9 studies lasted 9 weeks or less [38,49].

### Traumatic Brain Injury

This review found 9 studies [55-63] meeting the inclusion criteria for carers of people with traumatic brain injury (TBI; Multimedia Appendix 6). Of these studies, 8 featured interventions aimed specifically at carers of children and adolescents with TBI. All of these featured self-guided, modular interventions, followed by either a videoconference or Skype session with a therapist. Interventions varied in duration from 10 days [55] to 6 months [56-60], although a number of studies did not specify the length of the intervention [61-63]. Findings with regard to the effectiveness of these interventions were positive, with only 1 of the 9 studies failing to find an association between the intervention and positive carer outcomes [55].

## Discussion

### Principal Findings

This systematic review identified 46 studies investigating internet-based interventions for carers of individuals with psychiatric disorders, stroke, dementia, or TBI, with regard to their efficacy or effectiveness in improving or maintaining a range of facets of carer mental health. Findings for each of the different disorders are discussed below.

### Psychiatric Disorders

A total of 12 studies investigated the impact of an internet-based intervention on carers of individuals with a range of psychiatric disorders, including AN, depression, schizophrenia, ADHD, and mixed mental health difficulties. Individuals with these different disorders present with varying needs and support requirements, thus presenting different challenges for their carers, making it hard to compare across disorders. Moreover, study and intervention design varied considerably across studies, both of which potentially affect outcomes. Aspects of an intervention that may influence the impact of a specific intervention include the following: whether the intervention is theory-driven and based on a specific model of carer distress or, alternatively, how else the intervention content and format were decided upon (eg, focus groups, literature reviews, or clinician and researcher opinion), whether the intervention is delivered via a website or other internet-based technology (such as videoconferencing), whether the intervention is modular and sequential or allows unstructured exploration of a website, the extent to which the intervention is interactive (eg, is feedback given to participants on their knowledge, symptoms, or any other characteristics), whether and how it is supported, (eg, can participants interact with one another or communicate with clinicians and researchers), and, in addition, does the intervention contain elements other than text, such as video or audio features. Given the small number of studies available, there is a clear need for additional research. Possible future research directions are considered for each disorder below.

#### Anorexia Nervosa

Both studies concerning carers of individuals with AN tested aspects of the same intervention (OAO), which was derived from a model of carer distress, coproduced with carers, experts by experience, and professionals within the field [64]. In the first of these, the internet-based intervention was delivered with support from experienced clinicians, and improved carer distress was compared with usual support [20]. In the second study, which was small and underpowered, the addition of limited support by a trainee psychologist had no advantage over a internet-based intervention alone [21]. Taken together, these findings demonstrate that OAO can provide benefits for carer mental health, over and above the support typically offered to carers of individuals with AN. However, the impact of additional clinician support is currently unclear. This may be partly because of factors pertaining to the study methodology, including small sample size (n=37) and guidance being provided by trainee clinicians with limited experience in the field of eating disorders. Furthermore, the follow-up period (3-months) may be too short for carers to have fully honed and applied the skills taught within the program, given the chronic nature of AN, a carer’s behavior patterns may be even more long standing and ingrained than those found in other disorders. Future research in this area should seek to establish the additional benefit (if any) of clinician support and establish whether any observed differences were maintained at longer-term follow-up. In addition, it would also be useful to extend this work to carers of individuals diagnosed with other eating disorders, such as bulimia nervosa or binge eating disorder.

#### Depression

Only 1 study [22] assessed the efficacy of an internet-based intervention for carers of people with depression. The intervention was interactive and modular and based on psychoeducation and CBT techniques. Despite high reported levels of user friendliness, the intervention was ineffective. Of note, over 50% of participating carers kept their study participation a secret from those they cared for. Possible interpretations of this include carers not wishing it to be known that they require support, either as they fear the person they support may feel upset or guilty or as they wish to appear strong toward their loved one; carers not thinking it relevant or useful to share their participation with their loved one; carers not being sufficiently engaged with the intervention to treat it as an important program worthy of sharing and discussion. Research on the reasons carers do or do not share their involvement with internet-based support programs may be beneficial. Regardless of the reason(s), it is possible that this degree of secrecy contributed to the lack of efficacy of the intervention, as it may have made it harder for participants to alter their behavior and apply skills learned via the internet-based package (without the person they support finding out that they have accessed support), and it may thus inadvertently maintain the illness and carers’ own distress. Future interventions for carers of people with depression may need to address and remedy these issues.

#### Schizophrenia and Schizoaffective Disorder

A total of 2 studies focused on carers of individuals with schizophrenia; 1 was a small underpowered RCT of moderate quality [23], and the other employed a quasi-experimental design [24], rated to be of moderate quality.

The intervention reported in the Rotondi et al’s study [23] comprised a website, with the content aimed at both the individual with schizophrenia and the individual’s carer. Although this is an interesting idea, it would be important to establish whether an intervention based on an evidence-based model of distress specifically targeted at carers is more effective than one targeting both the individual with schizophrenia and the individual’s carer, as these 2 groups may have different needs. Furthermore, although both interventions permitted interaction with fellow participants, neither employed a modular approach. Research in other mental health populations found modular treatment to result in better outcomes compared with standard treatment [65]. Thus, it may be helpful for future research in this area to establish whether a more structured approach may be also associated with improvements in carer mental health. In addition, it may be helpful to establish whether the observed improvements in family relations may lead to improvements in carer mental health, if they are maintained over a longer period of time.

#### Attention-Deficit Hyperactivity Disorder

Neither of the 2 studies of interventions for carers of individuals with ADHD were RCTs; in a small case series [18], the group Triple P Parenting Program showed promise in reducing carer distress. A second study [25] involved a subset of data drawn from a larger study comparing the same intervention face to face or delivered via teletherapy. Carer distress only improved in the face-to-face delivery group. Although these findings are promising, RCTs are needed to further elucidate the role of internet-based interventions in supporting carers of people with ADHD. Qualitative data from carers may help explore carers’ views on the relative merits of face-to-face versus internet-based interventions, as in the Tse et al study [25], only carers receiving in-person training displayed improvements in their reported stress levels, although the content was the same in the internet-based intervention. Interventions in both studies appear to be based on behavioral and developmental models relating to ADHD; however, it may be helpful for future research to establish whether videoconferencing is the most effective way to deliver training to carers (in comparison, eg, with a website, where carers are able to work through and revisit the materials at their own pace).

#### Mixed Mental Health Difficulties

A total of 5 studies featured internet-based interventions for carers of individuals with mixed mental health difficulties, 3 of which [26,28,30] were RCTs, and 2 were prepost comparisons [27,29], with some evidence of reducing carer distress. These interventions were either codesigned with potential intervention users [29,30] or based pragmatically on use of mindfulness [26-28]—a technique that has been widely utilized across a range of population groups, but on the other hand, it is a technique that may not address the difficulties specific to the caring role; nonetheless, it was effective here. In future, it may be useful to compare disorder-specific carer interventions with more generic ones, potentially applicable to carers of people with a broad range of disorders to assess their relative merits in relation to their ability to improve carer outcomes.

### Stroke

A total of 2 of the 4 studies providing a internet-based intervention for carers of stroke survivors were moderate- and high-quality RCTs, respectively [32,33], with the remaining 2 studies [31,34] comprising relatively small, low-quality prepost comparison studies. We can only speculate on the reasons for this disparity in findings. The 2 studies [31,33] that found a positive impact of their interventions on carer mental health both delivered information to participants in a sequential, modular way. Such relatively structured intervention may be more accessible for older carers, who may have less experience in navigating websites. Of the studies that failed to find intervention effects on carer mental health, 1 [34] included carers of individuals with dementia, as well as carers of stroke survivors, which may have resulted in the content of the intervention being less tailored to the specific needs of stroke carers. Of note, both of the studies that did not show intervention effects in relation to carer mental health had other positive effects. In 1 of these, carers perceived greater social support following the internet-based intervention [34]. In the other study, stroke survivors whose carer received a internet-based intervention required fewer emergency department visits and fewer hospital readmissions than those whose carer did not, reducing the burden on the health service [32], which perhaps explains the lack of improvement in carer mental health. Further research in this area seems pressing, as in recent years, the number of stroke survivors has increased [66], with the vast majority of them living at home [67]. The informal carers of stroke survivors have been found to have high levels of depressive symptoms [68] and burden [69]. Thus, there is a considerable need for innovative interventions to support this population of carers. Future research in this area should seek to explore which particular aspect of a internet-based intervention leads to specific improvement across a wide variety of carer outcomes.

### Dementia

A total of 22 studies were found to investigate the effectiveness of internet-based interventions for carers of individuals with dementia (more than any other disorder discussed within this review), reflecting the size of this growing problem [70], the severity and range of behavioral and psychological symptoms that carers have to deal with in their loved one [71], and the corresponding severity of carer distress [14]. Findings from this review are largely in line with previous systematic reviews focused specifically on internet-based interventions for carers of people with dementia [72,73]. Although the results are mixed and this area of study would greatly benefit from more high-quality research, the evidence suggests that internet-based interventions may be useful in improving carer well-being and mental health.

There may be a number of reasons why these studies found mixed results regarding carer mental health; it may be the case that, over time, as the dementia sufferers’ condition worsens, their carer experiences a greater sense of burden and related symptoms of mental distress. Alternatively, acquiring increased knowledge about the typical symptoms, course, and progressive nature of dementia through a internet-based intervention may negatively affect carers’ mental health, especially if this is not buffered by having sufficient opportunity to have sensitive in-person discussions with a health professional. It is also of interest to note the disparate methods by which the interventions for dementia carers were developed. Although some appeared to be based to some extent on existing theories of, for example, stress and coping [35,74,75] or the transitions theory [39,50,76], the content of others was derived from interviews with carers and reviews of the relevant literature [48]. Future research in this area may want to investigate the feasibility of developing a model of carer distress specific to those supporting someone with dementia, on the basis of existing theories, in addition to being coproduced with carers. People with dementia are often cared for by their older spouses. For example, in a study of over 3800 dementia carers, the average age was found to be 63.3 years [77]. Thus, these carers may be less familiar with internet usage than younger people. As of 2011, only 41% of adults aged 65 years and above used the internet, in comparison to 94% of adults aged 18-29 years [78]. In addition, they may have visual or hearing impairments. Therefore, it is important to consider the appropriateness of internet-based interventions for this particular population and how such internet-based interventions may benefit from being altered to fit the specific needs of older carers. Several of the studies in this review used videoconferencing technology as part of their intervention—future research may wish to establish whether being able to see other carers or clinicians is more helpful to this population than interacting with a computer screen alone, as findings from this review are inconclusive with regard to this issue.

As noted above, there is some evidence that shorter interventions appeared to be more effective than those of longer duration in terms of their ability to reduce carer distress. The apparent effectiveness of shorter interventions in comparison to those of longer duration may have something to do with the nature of the illness. Over the course of the longer interventions, the symptoms of the person with dementia are likely to worsen, causing the person’s carer to experience elevated levels of stress and burden.

### Traumatic Brain Injury

We identified 9 studies, all of which were RCTs of either moderate or high quality, evaluating a internet-based intervention for carers of people with TBI. A total of 8 of these 9 studies found some positive impact of the intervention on carer mental health. The majority of the TBI-carer interventions identified comprised family problem solving therapy. Thus, these interventions may have been more homogenous, compared with those developed for carers of people with other psychiatric or neurological disorders (where interventions targeting the same populations have been developed from a wider range of sources, models, and theories). Furthermore, 8 of the 9 studies followed a similar structure, comprising self-guided, modular interventions, accompanied by internet-based interaction with a therapist. In future, it would be useful to establish whether either or both of these aspects—a structured, modular program (as opposed to a site containing links, which the carer is required to navigate without guidance) or support by a clinician—are particularly associated with more positive psychological outcomes in carers. As most current studies on carer interventions for people with TBI have focused on children and adolescents, future research should seek to address the needs of carers of adults with TBI.

### General Discussion

The field of internet-based mental health interventions is still relatively new, and it continues to develop rapidly, including the recent recognition of the potential of internet-based interventions specifically aimed at carers. A previous review of “telehealth” (video, internet-based, telephone based, and telemetry or remote monitoring) interventions for family carers found that a majority of interventions were satisfactory to carers, and they were associated with significant improvements to carer outcomes [79], indicating the possible viability and effectiveness of technology-based support or training. This review specifically explored the effectiveness of internet-based interventions, and it discovered largely mixed findings with regard to the impact of interventions on carer mental health and well-being, with the exception of those aimed at carer of children and adolescents with TBI, almost all of which were found to have a positive impact on carer outcomes. Studies in this review focus on carers of individuals with a range of difficulties, including psychiatric disorders, neurological disorders, and brain injury, with diverse challenges for carers. Therefore, unsurprisingly, the format, content, and nature of interventions used here vary widely. Nonetheless, the evidence-based relating to some areas (dementia, TBI) is more extensive (with 6 and 5 large-scale RCTs, >100 participants, respectively) than in other areas (stroke, psychiatric disorders), where fewer large-scale RCTs have been conducted. Across almost all areas (with TBI a notable exception), findings in relation to reductions of carer distress are somewhat mixed. This may largely have to do with differences in populations and aspects of study design. In addition, not all of the studies presented focused primarily on carer outcomes, and several interventions with little or no impact on carer distress had other benefits (eg, improvements in patient outcomes).

Currently, there is not enough evidence to conclude whether interventions specifically designed with a particular disorder in mind, based on a clear model of carer distress, have advantages over more generic interventions. Of note, in the area of TBI, many of the successful interventions utilized a problem-solving approach, and in the area of mixed mental health problems, mindfulness approaches were successfully used.

A further aspect of internet-based interventions for which the evidence is also currently mixed is the impact of guidance or support. Several interventions included in this review include some guidance; however, this varied widely among studies, making it difficult to draw conclusions about whether guided interventions are superior to nonguided ones and which aspects of guidance (mode of delivery, training, or expertise of guides) are most important. A previous systematic review found guidance to be a beneficial aspect of internet-based interventions for mental health; however, methodological issues and the small number studies included in the review make it difficult to analyze these findings [80]. When considering the effectiveness of internet-based interventions, it is important to consider how an intervention delivered via the internet may differ from face-to-face treatment. Surprisingly, this review found only 2 studies that directly compared internet-based with face-to-face interventions. In a small study (n=37), carers of children with ADHD who received face-to-face training were found to have improvements in their levels of stress and strain, in comparison to those receiving the same content delivered on the Web [25]. Carers of individuals with mild dementia who received a internet-based intervention were found to have significant improvements in their quality of life in comparison to those who received care as usual (specified as “infrequent counseling”), but no difference was found between the 2 groups on the other 3 relevant outcome measures [40]. Multiple studies reported control groups that received “care as usual,” but they did not specify what this comprised. Owing to the very small number of studies that did report a comparison between internet-based and face-to-face treatment, it is not possible to draw conclusions about how they compare in terms of impact on carer mental health or whether there are particular subgroups of carers who may benefit from one as opposed to the other. When attempting to explore the differences in response to the interventions discussed within this review, it may also be helpful to consider the possible applicability of Caregiver Identity Theory [7] to the responses of individual carers. Carer Identity Theory hypothesizes that the most significant factor influencing levels of distress is the disparity between carers’ responsibilities and their perceived identity standard. If this is indeed the case, then it may be important to assess the individual carer, the carer’s perceived burden, and the carer’s changing relationship with the person for whom they care to determine what type and intensity of intervention may most benefit them. A further possible factor in explaining the differences observed in the efficacy of the different Web interventions presented here may be the wide range of initiatives encompassed by the phrase “internet-based” (eg, interactive websites accessed in the carers’ own home vs videoconferencing chat, accessed from a local clinic). For some of the older studies included in this review, the technology utilized to provide the intervention may now be considered to be out of date. As the development of technology continues at an increasing pace, what may currently constitute a typical internet-based intervention may appear outdated in just a few years.

### Limitations

Several key limitations need to be noted. Although the majority of studies (29/46) were RCTs, a significant proportion comprised single-arm, prepost trials, with no randomization or control group comparisons. Quality varied widely across all studies, with some of the studies lacking key information on randomization methodology, blinding of assessors, and not having an accessible study protocol. This increases the difficulty of assessing the risk of bias within studies, and this makes it harder to interpret the effectiveness of the intervention reported. Overall, 16 RCTs and 0 non-RCTs were rated as high quality, 11 RCTs and 6 non-RCTs were rated as moderate quality, and 2 RCTs and 11 non-RCTs were rated as low quality, with study quality also varying across disorder type, making it hard to compare studies that were investigating the same type of disorder. Although we have reported all findings relating to carer mental health that were reported in the original study papers, a majority of the studies were not found to have a published paper detailing the study protocol, meaning that it was not always possible to confirm the absence of publication or selective reporting bias. Owing to the wide range of outcomes employed across studies to measure change in carer mental health and related constructs, we were unable to conduct any meta-analyses, making it more difficult to interpret and compare findings across studies. In addition, the diverse range of formats used to deliver the interventions and differing levels and types of guidance offered made it harder to compare the findings, and this may have had an impact on how engaging they are to carers.

### Future Considerations

Although the studies detailed above present a mixed picture regarding the overall effect of internet-based interventions on carer mental health, participating carers consistently reported that they found internet-based interventions to be highly acceptable and easy to utilize where these data were gathered. This may indicate that carers are willing to integrate internet-based interventions into their daily lives, and future research should attempt to establish the most effective way of delivering content to have the greatest impact possible on carer mental health. Of the 46 studies identified within this review, only 2 reported a direct comprising internet-based versus face-to-face interventions, of which only 1 study compared the same content delivered via the 2 different modalities. Future research should seek to establish whether the method of delivery of an intervention has an impact on carer outcomes and whether there are specific subgroups of carers who may benefit more from one than the other. As described above, internet-based interventions can comprise many different components, and it would be beneficial for future research to establish the degree to which particular elements contribute to forming an effective intervention. For example, it would be useful to be able to establish the active components of a given intervention; therefore, findings can be more easily accumulated and compared across interventions, improving the evidence base regarding which components of an intervention lead to desired behavior change [81]. The possibility of establishing specific models of carer distress on which interventions can be built should also be taken into consideration, in addition to consulting additional sources of information (such as focus groups, expert opinion, and reviews of the relevant literature). Future work into the impact of caring carer-interventions should consider taking a lifespan approach when considering the challenges of caring, both in terms of the carer life stage (eg, the differing requirements of young carers and older spousal carers) and that of the individual being cared for, as well as how these challenges can change and develop over time. Consideration (including with regard to cost effectiveness) should also be given to the possibility of blended care, where carers would receive some face-to-face clinician contact, in addition to accessing a internet-based intervention.

### Conclusions

The emerging field of internet-based interventions for carers of individuals with psychiatric disorders, neurological disorders, and brain injury offers exciting possibilities for providing support to a population that may otherwise find it difficult to access help, by giving them the option of accessing a range of relevant interventions in a more flexible way, or as part of a stepped-care program. Although findings from existing studies are mixed with regard to evidence of the efficacy of internet-based interventions, they show promise in terms of both effectiveness and acceptability, and further research into this area may establish the most effective ways in which internet-based interventions for carers can be utilized.

## Appendix

Multimedia Appendix 1 Risk of bias of included studies.

Multimedia Appendix 2 Summary of studies: carers of individuals with psychiatric disorders.

Multimedia Appendix 3 Summary of studies: carers of individuals who have survived a stroke.

Multimedia Appendix 4 Summary of studies: carers of individuals with dementia (studies with a control group).

Multimedia Appendix 5 Summary of studies: carers of individuals with dementia (studies without a control group).

Multimedia Appendix 6 Summary of studies: carers of individuals with traumatic brain injury.

## Abbreviations

ADHD: attention-deficit hyperactivity disorder
AN: anorexia nervosa
BRC: Biomedical Research Centre
CBT: cognitive behavioral therapy
NHS: National Health Service
NIHR: National Institute for Health Research
OAO: Overcoming Anorexia Online
RCT: randomized controlled trial
TBI: traumatic brain injury

## References

[1] Buckner L, Yeandle S (2015). Carers UK.

[2] Brodaty H, Green A, Koschera A (2003). Meta-analysis of psychosocial interventions for caregivers of people with dementia. J Am Geriatr Soc.

[3] Jungbauer J, Wittmund B, Dietrich S, Angermeyer MC (2004). The disregarded caregivers: subjective burden in spouses of schizophrenia patients. Schizophr Bull.

[4] Martin Y, Gilbert P, McEwan K, Irons C (2006). The relation of entrapment, shame and guilt to depression, in carers of people with dementia. Aging Ment Health.

[5] Baumgarten M, Battista RN, Infante-Rivard C, Hanley JA, Becker R, Gauthier S (1992). The psychological and physical health of family members caring for an elderly person with dementia. J Clin Epidemiol.

[6] Magliano L, Fiorillo A, De Rosa C, Malangone C, Maj M, National Mental Health Project Working Group (2005). Family burden in long-term diseases: a comparative study in schizophrenia vs physical disorders. Soc Sci Med.

[7] Montgomery R, Kosloski K (2009). Caregiving as a process of changing identity: implications for caregiver support. Generations.

[8] Yesufu-Udechuku A, Harrison B, Mayo-Wilson E, Young N, Woodhams P, Shiers D, Kuipers E, Kendall T (2015). Interventions to improve the experience of caring for people with severe mental illness: systematic review and meta-analysis. Br J Psychiatry.

[9] Blom MM, Bosmans JE, Cuijpers P, Zarit SH, Pot AM (2013). Effectiveness and cost-effectiveness of an internet intervention for family caregivers of people with dementia: design of a randomized controlled trial. BMC Psychiatry.

[10] Berger T, Hämmerli K, Gubser N, Andersson G, Caspar F (2011). Internet-based treatment of depression: a randomized controlled trial comparing guided with unguided self-help. Cogn Behav Ther.

[11] Robinson E, Titov N, Andrews G, McIntyre K, Schwencke G, Solley K (2010). Internet treatment for generalized anxiety disorder: a randomized controlled trial comparing clinician vs technician assistance. PLoS One.

[12] Knaevelsrud C, Maercker A (2007). Internet-based treatment for PTSD reduces distress and facilitates the development of a strong therapeutic alliance: a randomized controlled clinical trial. BMC Psychiatry.

[13] Sánchez-Ortiz VC, Munro C, Stahl D, House J, Startup H, Treasure J, Williams C, Schmidt U (2011). A randomized controlled trial of internet-based cognitive-behavioural therapy for bulimia nervosa or related disorders in a student population. Psychol Med.

[14] Pinquart M, Sörensen S (2003). Differences between caregivers and noncaregivers in psychological health and physical health: a meta-analysis. Psychol Aging.

[15] Hu C, Kung S, Rummans TA, Clark MM, Lapid MI (2014). Reducing caregiver stress with internet-based interventions: a systematic review of open-label and randomized controlled trials. J Am Med Inform Assoc.

[16] Ploeg J, Markle-Reid M, Valaitis R, McAiney C, Duggleby W, Bartholomew A, Sherifali D (2017). Web-based interventions to improve mental health, general caregiving outcomes, and general health for informal caregivers of adults with chronic conditions living in the community: rapid evidence review. J Med Internet Res.

[17] Cristancho-Lacroix V, Wrobel J, Cantegreil-Kallen I, Dub T, Rouquette A, Rigaud A (2015). A web-based psychoeducational program for informal caregivers of patients with Alzheimer's disease: a pilot randomized controlled trial. J Med Internet Res.

[18] Reese RJ, Slone NC, Soares N, Sprang R (2012). Telehealth for underserved families: an evidence-based parenting program. Psychol Serv.

[19] Brockmeyer T, Friederich H, Schmidt U (2018). Advances in the treatment of anorexia nervosa: a review of established and emerging interventions. Psychol Med.

[20] Grover M, Naumann U, Mohammad-Dar L, Glennon D, Ringwood S, Eisler I, Williams C, Treasure J, Schmidt U (2011). A randomized controlled trial of an Internet-based cognitive-behavioural skills package for carers of people with anorexia nervosa. Psychol Med.

[21] Hoyle D, Slater J, Williams C, Schmidt U, Wade TD (2013). Evaluation of a web-based skills intervention for carers of people with anorexia nervosa: a randomized controlled trial. Int J Eat Disord.

[22] Bijker L, Kleiboer A, Riper H, Cuijpers P, Donker T (2017). A pilot randomized controlled trial of E-care for caregivers: an internet intervention for caregivers of depressed patients. Internet Interv.

[23] Rotondi AJ, Haas GL, Anderson CM, Newhill CE, Spring MB, Ganguli R, Gardner WB, Rosenstock JB (2005). A clinical trial to test the feasibility of a telehealth psychoeducational intervention for persons with schizophrenia and their families: intervention and 3-month findings. Rehabil Psychol.

[24] Glynn SM, Randolph ET, Garrick T, Lui A (2010). A proof of concept trial of an online psychoeducational program for relatives of both veterans and civilians living with schizophrenia. Psychiatr Rehabil J.

[25] Tse YJ, McCarty CA, Stoep AV, Myers KM (2015). Teletherapy delivery of caregiver behavior training for children with attention-deficit hyperactivity disorder. Telemed J E Health.

[26] Stjernswärd S, Hansson L (2017). Effectiveness and usability of a web-based mindfulness intervention for families living with mental illness. Mindfulness (N Y).

[27] Stjernswärd S, Hansson L (2017). Outcome of a web-based mindfulness intervention for families living with mental illness - a feasibility study. Inform Health Soc Care.

[28] Stjernswärd S, Hansson L (2018). Effectiveness and usability of a web-based mindfulness intervention for caregivers of people with mental or somatic illness. A randomized controlled trial. Internet Interv.

[29] Gleeson J, Lederman R, Koval P, Wadley G, Bendall S, Cotton S, Herrman H, Crisp K, Alvarez-Jimenez M (2017). Moderated online social therapy: a model for reducing stress in carers of young people diagnosed with mental health disorders. Front Psychol.

[30] Ali L, Krevers B, Sjöström N, Skärsäter I (2014). Effectiveness of web-based versus folder support interventions for young informal carers of persons with mental illness: a randomized controlled trial. Patient Educ Couns.

[31] Graf R, LeLaurin J, Schmitzberger M, Freytes IM, Orozco T, Dang S, Uphold CR (2017). The stroke caregiving trajectory in relation to caregiver depressive symptoms, burden, and intervention outcomes. Top Stroke Rehabil.

[32] Pierce LL, Steiner VL, Khuder SA, Govoni AL, Horn LJ (2009). The effect of a web-based stroke intervention on carers' well-being and survivors' use of healthcare services. Disabil Rehabil.

[33] Smith GC, Egbert N, Dellman-Jenkins M, Nanna K, Palmieri PA (2012). Reducing depression in stroke survivors and their informal caregivers: a randomized clinical trial of a web-based intervention. Rehabil Psychol.

[34] Torp S, Hanson E, Hauge S, Ulstein I, Magnusson L (2008). A pilot study of how information and communication technology may contribute to health promotion among elderly spousal carers in Norway. Health Soc Care Community.

[35] Beauchamp N, Irvine AB, Seeley J, Johnson B (2005). Worksite-based internet multimedia program for family caregivers of persons with dementia. Gerontologist.

[36] Eisdorfer C, Czaja SJ, Loewenstein DA, Rubert MP, Argüelles S, Mitrani VB, Szapocznik J (2003). The effect of a family therapy and technology-based intervention on caregiver depression. Gerontologist.

[37] van Mierlo LD, Meiland FJ, van de Ven PM, van Hout HP, Dröes RM (2015). Evaluation of DEM-DISC, customized e-advice on health and social support services for informal carers and case managers of people with dementia; a cluster randomized trial. Int Psychogeriatr.

[38] Pagán-Ortiz ME, Cortés DE, Rudloff N, Weitzman P, Levkoff S (2014). Use of an online community to provide support to caregivers of people with dementia. J Gerontol Soc Work.

[39] Duggleby W, Ploeg J, McAiney C, Peacock S, Fisher K, Ghosh S, Markle-Reid M, Swindle J, Williams A, Triscott JA, Forbes D, Jovel Ruiz K (2018). Web-based intervention for family carers of persons with dementia and multiple chronic conditions (My Tools 4 Care): pragmatic randomized controlled trial. J Med Internet Res.

[40] Boots LM, de Vugt ME, Kempen GI, Verhey FR (2018). Effectiveness of a blended care self-management program for caregivers of people with early-stage dementia (partner in balance): randomized controlled trial. J Med Internet Res.

[41] Blom MM, Zarit SH, Zwaaftink RB, Cuijpers P, Pot AM (2015). Effectiveness of an Internet intervention for family caregivers of people with dementia: results of a randomized controlled trial. PLoS One.

[42] Czaja SJ, Loewenstein D, Schulz R, Nair SN, Perdomo D (2013). A videophone psychosocial intervention for dementia caregivers. Am J Geriatr Psychiatry.

[43] Kajiyama B, Thompson LW, Eto-Iwase T, Yamashita M, Di Mario J, Marian Tzuang Y, Gallagher-Thompson D (2013). Exploring the effectiveness of an internet-based program for reducing caregiver distress using the iCare Stress Management e-Training Program. Aging Ment Health.

[44] Marziali E, Donahue P (2006). Caring for others: internet video-conferencing group intervention for family caregivers of older adults with neurodegenerative disease. Gerontologist.

[45] Torkamani M, McDonald L, Aguayo IS, Kanios C, Katsanou MN, Madeley L, Limousin PD, Lees AJ, Haritou M, Jahanshahi M, ALADDIN Collaborative Group (2014). A randomized controlled pilot study to evaluate a technology platform for the assisted living of people with dementia and their carers. J Alzheimers Dis.

[46] Griffiths PC, Whitney MK, Kovaleva M, Hepburn K (2016). Development and implementation of tele-savvy for dementia caregivers: a department of veterans affairs clinical demonstration project. Gerontologist.

[47] Kwok T, Au A, Wong B, Ip I, Mak V, Ho F (2014). Effectiveness of online cognitive behavioral therapy on family caregivers of people with dementia. Clin Interv Aging.

[48] Lorig K, Thompson-Gallagher D, Traylor L, Ritter PL, Laurent DD, Plant K, Thompson LW, Hahn TJ (2010). Building better caregivers: a pilot online support workshop for family caregivers of cognitively impaired adults. J Appl Gerontol.

[49] Bateman DR, Brady E, Wilkerson D, Yi E, Karanam Y, Callahan CM (2017). Comparing crowdsourcing and friendsourcing: a social media-based feasibility study to support Alzheimer disease caregivers. JMIR Res Protoc.

[50] Duggleby W, Jovel Ruiz K, Ploeg J, McAiney C, Peacock S, Nekolaichuk C, Holroyd-Leduc J, Ghosh S, Brazil K, Swindle J, Forbes D, Woodhead Lyons S, Parmar J, Kaasalainen S, Cottrell L, Paragg J (2018). Mixed-methods single-arm repeated measures study evaluating the feasibility of a web-based intervention to support family carers of persons with dementia in long-term care facilities. Pilot Feasibility Stud.

[51] Chiu T, Marziali E, Colantonio A, Carswell A, Gruneir M, Tang M, Eysenbach G (2009). Internet-based caregiver support for Chinese Canadians taking care of a family member with alzheimer disease and related dementia. Can J Aging.

[52] Glueckauf RL, Ketterson TU, Loomis JS, Dages P (2004). Online support and education for dementia caregivers: overview, utilization, and initial program evaluation. Telemed J E Health.

[53] Lindauer A, Croff R, Mincks K, Mattek N, Shofner SJ, Bouranis N, Teri L (2018). "It Took the Stress out of Getting Help": The STAR-C-Telemedicine Mixed Methods Pilot. Care Wkly.

[54] Marziali E, Garcia LJ (2011). Dementia caregivers' responses to 2 Internet-based intervention programs. Am J Alzheimers Dis Other Demen.

[55] McLaughlin KA, Glang A, Beaver SV, Gau JM, Keen S (2013). Web-based training in family advocacy. J Head Trauma Rehabil.

[56] Petranovich CL, Wade SL, Taylor HG, Cassedy A, Stancin T, Kirkwood MW, Maines Brown T (2015). Long-term caregiver mental health outcomes following a predominately online intervention for adolescents with complicated mild to severe traumatic brain injury. J Pediatr Psychol.

[57] Raj SP, Antonini TN, Oberjohn KS, Cassedy A, Makoroff KL, Wade SL (2015). Web-based parenting skills program for pediatric traumatic brain injury reduces psychological distress among lower-income parents. J Head Trauma Rehabil.

[58] Raj SP, Shultz EL, Zang H, Zhang N, Kirkwood MW, Taylor HG, Stancin T, Yeates KO, Wade SL (2018). Effects of web-based parent training on caregiver functioning following pediatric traumatic brain injury: a randomized control trial. J Head Trauma Rehabil.

[59] Wade SL, Walz NC, Carey J, McMullen KM, Cass J, Mark E, Yeates KO (2012). A randomized trial of teen online problem solving: efficacy in improving caregiver outcomes after brain injury. Health Psychol.

[60] Wade SL, Karver CL, Taylor HG, Cassedy A, Stancin T, Kirkwood MW, Brown TM (2014). Counselor-assisted problem solving improves caregiver efficacy following adolescent brain injury. Rehabil Psychol.

[61] Carey JC, Wade SL, Wolfe CR (2008). Lessons learned: the effect of prior technology use on web-based interventions. Cyberpsychol Behav.

[62] Wade SL, Carey J, Wolfe CR (2006). An online family intervention to reduce parental distress following pediatric brain injury. J Consult Clin Psychol.

[63] Wade SL, Walz NC, Carey JC, Williams KM (2008). Preliminary efficacy of a web-based family problem-solving treatment program for adolescents with traumatic brain injury. J Head Trauma Rehabil.

[64] Treasure J, Whitaker W, Whitney J, Schmidt U (2005). Working with families of adults with anorexia nervosa. J Family Therapy.

[65] Weisz JR, Chorpita BF, Palinkas LA, Schoenwald SK, Miranda J, Bearman SK, Daleiden EL, Ugueto AM, Ho A, Martin J, Gray J, Alleyne A, Langer DA, Southam-Gerow MA, Gibbons RD, Research Network on Youth Mental Health (2012). Testing standard and modular designs for psychotherapy treating depression, anxiety, and conduct problems in youth: a randomized effectiveness trial. Arch Gen Psychiatry.

[66] Feigin VL, Forouzanfar MH, Krishnamurthi R, Mensah GA, Connor M, Bennett DA, Moran AE, Sacco RL, Anderson L, Truelsen T, O'Donnell M, Venketasubramanian N, Barker-Collo S, Lawes CM, Wang W, Shinohara Y, Witt E, Ezzati M, Naghavi M, Murray C, Global Burden of Diseases‚ Injuries‚ Risk Factors Study 2010 (GBD 2010) the GBD Stroke Experts Group (2014). Global and regional burden of stroke during 1990-2010: findings from the Global Burden of Disease Study 2010. Lancet.

[67] Crichton SL, Bray BD, McKevitt C, Rudd AG, Wolfe CD (2016). Patient outcomes up to 15 years after stroke: survival, disability, quality of life, cognition and mental health. J Neurol Neurosurg Psychiatry.

[68] Visser-Meily A, Post M, van de Port I, Maas C, Forstberg-Wärleby G, Lindeman E (2009). Psychosocial functioning of spouses of patients with stroke from initial inpatient rehabilitation to 3 years poststroke: course and relations with coping strategies. Stroke.

[69] Jaracz K, Grabowska-Fudala B, Górna K, Jaracz J, Moczko J, Kozubski W (2015). Burden in caregivers of long-term stroke survivors: Prevalence and determinants at 6 months and 5 years after stroke. Patient Educ Couns.

[70] Prince M (2015). Alzheimer's Disease International.

[71] Savva GM, Zaccai J, Matthews FE, Davidson JE, McKeith I, Brayne C (2009). Prevalence, correlates and course of behavioural and psychological symptoms of dementia in the population. Br J Psychiatry.

[72] Boots LM, de Vugt ME, van Knippenberg RJ, Kempen GI, Verhey FR (2014). A systematic review of internet-based supportive interventions for caregivers of patients with dementia. Int J Geriatr Psychiatry.

[73] Hopwood J, Walker N, McDonagh L, Rait G, Walters K, Iliffe S, Ross J, Davies N (2018). Internet-based interventions aimed at supporting family caregivers of people with dementia: systematic review. J Med Internet Res.

[74] Lazarus RS, Folkman S (1984). Stress, Appraisal, And Coping.

[75] Gallagher-Thompson D, Solano N, McGee JS, Krisztal E, Kaye J, Coon DW, Thompson LW (2002). Coping With Caregiving: Reducing Stress and Improving Your Quality of Life.

[76] Meleis A (2015). Transitions Theory. Nursing Theories And Nursing Practice.

[77] Yaffe K, Fox P, Newcomer R, Sands L, Lindquist K, Dane K, Covinsky KE (2002). Patient and caregiver characteristics and nursing home placement in patients with dementia. J Am Med Assoc.

[78] Zickuhr K, Smith A (2012). Pew Internet.

[79] Chi N, Demiris G (2015). A systematic review of telehealth tools and interventions to support family caregivers. J Telemed Telecare.

[80] Baumeister H, Reichler L, Munzinger M, Lin J (2014). The impact of guidance on Internet-based mental health interventions — a systematic review. Internet Interv.

[81] Michie S, Richardson M, Johnston M, Abraham C, Francis J, Hardeman W, Eccles MP, Cane J, Wood CE (2013). The behavior change technique taxonomy (v1) of 93 hierarchically clustered techniques: building an international consensus for the reporting of behavior change interventions. Ann Behav Med.

