# Supplementary material for: Internet-Based Interventions for Carers of Individuals With Psychiatric Disorders, Neurological Disorders, or Brain Injuries: Systematic Review
Source: J Med Internet Res. 2019 Jul 9;21(7):e10876. doi: 10.2196/10876 (PMC6647754; doi:10.2196/10876)
Supplement: Multimedia Appendix 1 [file jmir_v21i7e10876_app1.pdf]

Multimedia Appendix 1: Risk of Bias of Included Studies

**Quality Item**																					
	1	2	3	4	5	6	7	8	9	10	11	12	13	14	15	16	17	18	19	20	Total
**Study Reference**																					
Psychiatric Disorders																					
[20]	●	●	●	●	●	●		●	●	●	●	●		●	●	●	●	●	●	●	18/20
[21]	●			●	●	●		●	●	●				●		●	●	●	●	●	13/20
[22]	●	●		●	●		●	●	●	●	●			●	●	●		●	●	●	15/20
[24]				●	■	●	■	●	●	■	■	■	■	■		●		●	●	●	8/13
[23]	●			●	●		●	●							●	●	●	●	●		10/20
[18]					■		■	●	●	■	■	■	■	■					●	●	4/13
[25]					■		■	●	●	■	■	■	■	■	●	●			●	●	6/13
[30]	●	●	●	●	●		●	●	●	●	●			●	●			●	●	●	15/20
[29]				●	■		■		●	■	■	■	■	■	●			●	●	●	6/13
[26]	●	●	●	●	●			●	●	●	●			●		●		●	●	●	14/20
[27]		●		●	■	●	■		●	■	■	■	■	■				●	●	●	7/13
[28]	●	●	●	●	●		●	●	●	●	●			●				●	●	●	14/20
Stroke																					
[31]		●		●	■		■		●	■	■	■	■	■				●	●	●	6/13
[32]	●	●	●	●	●			●	●					●		●	●	●	●	●	13/20
[33]	●			●	●	●	●	●	●	●	●	●		●				●	●	●	14/20
[34]				●	■	●	■		●	■	■	■	■	■				●	●	●	6/13
Dementia – Studies with a Control Group																					
[35]	●	●		●	●	●		●	●					●				●	●	●	11/20
[71]	●	●		●	●		●	●	●	●	●	●	●	●	●				●	●	15/20
[68]	●	●		●	●	●			●	●	●	●		●	●	●		●	●	●	15/20
[17]	●		●	●			●	●	●	●	●			●	●	●		●	●	●	14/20
[72]	●	●		●	●	●		●	●					●				●	●	●	11/20
[62]	●	●		●	●		●	●	●	●			●	●	●	●		●	●	●	15/20
[36]	●	●		●					●					●				●	●	●	8/20
[74]	●	●		●	●			●	●					●	●	●		●	●	●	12/20
[76]	●	●		●	●				●					●		●		●	●	●	10/20
[41]					■		■	●	●	■	■	■	■	■					●	●	4/13
[77]	●	●		●				●	●									●	●	●	8/20
[37]	●	●	●	●		●	●	●	●	●	●			●				●	●	●	14/20
Dementia – Studies without a Control Group																					
[42]				●	■		■		●	■	■	■	■	■				●	●	●	5/13
[78]					■		■	●	●	■	■	■	■	■					●	●	4/13
[63]		●		●	■	●	■		●	■	■	■	■	■	●			●	●	●	8/13
[79]					■		■	●	●	■	■	■	■	■		●			●	●	5/13
[38]				●	■		■	●	●	■	■	■	■	■					●	●	5/13
[39]		●		●	■		■	●	●	■	■	■	■	■		●		●	●	●	8/13
[80]					■	●	■		●	■	■	■	■	■		●			●	●	5/13
[40]		●		●	■		■	●	●	■	■	■	■	■	●	●		●	●	●	9/13
[81]		●		●	■		■	●	●	■	■	■	■	■		●		●	●	●	8/13
[34]				●	■	●	■		●	■	■	■	■	■				●	●	●	6/13
Traumatic Brain Injury (TBI)																					
[49]	●			●	●	●		●	●	●							●	●	●	●	11/20
[43]	●	●		●	●		●	●	●	●						●	●	●	●		12/20
[44]	●	●		●	●			●	●	●	●	●		●	●	●	●	●	●	●	16/20
[45]	●			●	●	●		●	●	●	●			●	●		●	●	●	●	14/20
[46]	●	●	●	●	●			●	●	●				●	●	●		●	●	●	14/20
[50]	●			●	●	●	●	●	●	●	●			●				●	●	●	13/20
[71]	●			●		●		●	●	●	●			●	●			●	●	●	12/20
[47]	●			●	●	●		●	●	●	●			●	●	●	●	●	●	●	15/20
[48]	●	●		●	●	●	●	●	●	●	●	●		●	●	●		●	●	●	17/20

Quality Item Criteria List

1. Study utilises RCT design
2. Sample size n > 30 in each condition
3. A priori power analysis conducted
4. No recruitment (selection) bias
5. Groups similar at baseline
6. Drop-out rate < 20%
7. ITT Analysis
8. All relevant outcomes reported
9. Validated, reliable outcome measures
10. Adequate method of randomisation
11. Allocation concealment (for RCTs)
12. Blinding of assessors
13. Blinding of participants and treatment providers
14. Consort statement (drop-outs reported)
15. Registered in clinical trial registry
16. Good adherence to intervention protocols
17. Other interventions avoided, or similar in conditions
18. Population representative (relevant)
19. Intervention relevant
20. Primary endpoint clinically relevant
