# Supplementary material for: Internet-Based Interventions for Carers of Individuals With Psychiatric Disorders, Neurological Disorders, or Brain Injuries: Systematic Review
Source: J Med Internet Res. 2019 Jul 9;21(7):e10876. doi: 10.2196/10876 (PMC6647754; doi:10.2196/10876)
Supplement: Multimedia Appendix 2 [file jmir_v21i7e10876_app2.pdf]

Multimedia Appendix 2: Summary of studies – carers of individuals with psychiatric disorders

Participants and Study Reference	Study Design, Timeline, and Quality	Web-based intervention	Comparison / Control Group	Findings	Comments
64 carers of individuals with anorexia nervosa (AN). Intervention (n = 33), mean age = 47.3, 69.7% mothers. Control (n = 30), mean age = 49.1, 90% mothers). [20]	RCT<br><br>*Timeline* – Baseline, 4 months, 6 months.<br><br>*Study Quality* = High	Overcoming Anorexia Online (OAO) web intervention.<br><br>*Model / Development* – ED carers distress model. CBT and systemic framework.<br>*Interactivity* – Moderated message boards to contact other carers. Weekly phone/email contact with clinician.<br>*Structure* – 8 sequential modules.<br>*Duration* – 4 months.	'Beat' charity support as usual. Including telephone hotline, email support, support groups, moderated message board.	***Depression and anxiety*** **(HADS)** – additional benefit of OAO found to reduce HADS score (*p*=0.033, 95% CI [-7.3, -0.3] in comparison with control group (pre-post treatment).<br><br>*Effectiveness of Intervention score =**3**.*	Additional measures (e.g. eating disorder symptomatology, expressed emotion) showed greater reduction in OAO compared to the control group, but these were not significant.
37 carers of individuals with AN. OAO + guidance, n = 19), OAO alone, n = 18. Mean age not reported, 89.2% female. [21]	RCT<br><br>*Timeline* – Baseline, post-intervention (7 weeks), 3 months.<br><br>*Study Quality* = Moderate	Overcoming Anorexia Online (OAO) web intervention.<br><br>*Model / Development* – ED carers distress model. CBT and systemic framework.<br>*Interactivity* – Moderated message boards to contact other carers. Weekly phone or email contact with clinician.<br>*Structure* – 7 sequential modules.<br>*Duration* – 7 weeks.	Overcoming Anorexia Online (OAO) with no additional guidance.	*Depression, anxiety, and stress* (DASS) – no significant differences in either group over time.<br>*GHQ-28* - no significant differences in either group over time.<br><br>*Effectiveness of Intervention score = **1***	Some significant findings over other measures (negative experiences of caring, intrusiveness, impact of starvation) – findings mixed regarding whether clinician guidance improves scores.<br>**Expressed emotion** as perceived by the individual with AN not found to change over time.
80 carers of individuals with depression. Intervention (n = 41), mean age = 50, 80.5% female. Control (n = 39), mean age = 49.8, 74.4% female. [22]	RCT<br><br>*Timeline* – Baseline, post-intervention (6 weeks).<br><br>*Study Quality* = High	E-care for caregivers<br><br>*Model / Development* – based on self-help manual for carers of depressed people, based on focus groups. Psychoeducation and CBT techniques.<br>*Interactivity* – Personalised feedback from trained CBT coach, mediated internet forum (Facebook group).<br>*Structure* - 8 non-sequential modules.<br>*Duration* – 6 weeks	Waitlist control	***Psychological distress*** **(K10)** – No significant difference between groups.<br>*Anxiety* (GAD-7) – No significant difference between groups.<br>*Burden* (ZBI) – No significant difference between groups.<br>*QoL* (EQ5D) - No significant difference between groups.	User friendliness assessed by SUS; 'good and stable' (average score of 81.5 / 100).<br>Over half of carers expressed that they wished to keep their loved one unaware of their participation, due to not wanting them to feel guilt that they are seeking support.

				*Effectiveness of Intervention score = **1***	
42 families of individuals with schizophrenia. Intervention (n = 26); mean age = 57, 77% female. Control (n = 16); mean age = 53.8, 94% female. [24]	Quasi-experimental<br><br>*Timeline* – Baseline, 6 months, 12 months.<br><br>*Study Quality* = Moderate	Online relative support program with resource links.<br><br>*Model / Development* – Refers to diathesis-stress model, video presentations from book on family therapy<br>*Interactivity* – Facilitated real-time chat (closed cohorts of 5 or 6), discussion board, video presentations.<br>*Structure* – Non-modular online support program.<br>*Duration* – 12 months.	Usual care – access to all typical available services. Experimenters did not attempt to influence care received.	*Distress* (BSI) – No significant differences between groups on measured subscales.<br><br>*Effectiveness of Intervention score =**1***	No differences in BPRS scores in individuals with schizophrenia. Family relationship stress improved over time in online group.<br>Majority of users satisfied with online program.
21 support persons of individuals with schizophrenia / schizoaffective disorder. Intervention n = 11, control n = 10. Mean age = 51.52, 66.7% female. [23]	RCT<br><br>*Timeline* – Baseline, 3 months.<br><br>*Study Quality* = Moderate	Telehealth intervention: The Schizophrenia Guide website. Library of answered questions, activities, news, and educational materials.<br><br>*Model / Development* – Refers to diathesis-stress, stress-coping, and family psychoeducational models. Topic content based on interviews, previous work by the authors, needs assessments, and advisory board guidance.<br>*Interactivity* – Facilitated online therapy groups, Q&A with experts.<br>*Structure* – Non-modular website.<br>*Duration* – 3 months.	Care as usual.	*Stress* – No differences in carer perceived stress between two groups at 3 months.<br><br>*Effectiveness of Intervention score =**1***	Almost all (90.9%) family members / support persons indicated that they would like to be involved in a family / support person therapy group if this was to be offered after the conclusion of the study.
8 families of children with ADHD. Mean age = 33.8, 71.4% female. [18]	Pre-post comparison<br><br>*Timeline* – Not reported.<br><br>*Study Quality* = Low	Group Triple P Positive Parenting Program<br><br>*Model / Development* – behavioural psychoeducation programme, drawn from social learning and developmental pathology models, and family behaviour therapy.<br>*Interactivity* – Unclear; delivered by videoconferencing technology at regional medical centre.	n/a	*Depression, anxiety, and stress* (DASS) – parental distress decreased from pre-post (d=-0.34).<br><br>*Effectiveness of Intervention score =**3***	Video-conferencing technology utilising internet connection, as opposed to website-based intervention. Carers required to come to regional medical centre. Not all quantitative data reported.

		Structure – 8, non-modular videoconferencing sessions<br>*Duration* – Not reported.			
37 families of children with ADHD. Teletherapy group n = 12, in-person training group n = 25. [25]	Unclear – larger CATTS trial was an RCT; not specified how this sub-sample was chosen<br><br>*Timeline* – Baseline, 25 weeks.<br><br>*Study Quality* = Low	Children's ADHD Telemental Health Treatment Study (CATTS) intervention; Pharmacotherapy with caregiver behaviour training intervention<br><br>*Model / Development* – Carer behaviour training, based on reviews of evidence base for treating youth with ADHD.<br>*Interactivity* – Training via video-teleconferencing.<br>*Structure* – 6 sessions, 3-4 weeks apart.<br>*Duration* – 25 weeks.	Same behaviour training, delivered face-to-face.	*Depression* (PHQ-9) – No significant improvement in either group.<br>*Stress* (PSI) – Improvement in face-to-face group over time; ($p<0.05$), but not teletherapy.<br>*Strain* (CGSQ) - Improvement in face-to-face group over time; ($p<0.01$), but not teletherapy.<br><br>*Effectiveness of Intervention score =**1***	Families showed comparable attendance, satisfaction, and improvement in their child's level of functioning with regards to scores on the **VADRS ADHD diagnostic scale.**
241 young carers (age 16-25; mean = 20.5) of individuals with a range of mental health difficulties. Web-based support n = 120, folder support n = 121. 70.99% female. [30]	RCT<br><br>*Timeline* – Baseline, 4 months, 8 months.<br><br>*Study Quality* = High	Website support with asynchronous information, advice, self-care tips, where and when to seek help.<br><br>*Model / Development* – Developed in collaboration with young informal carers of a person with mental illness.<br>*Interactivity* – Forum for participants to have real-time discussions. Able to send questions (published anonymously in FAQs) to support team.<br>*Structure* – Non-modular website.<br>*Duration* – Not detailed. T2 at 4 months.	Folder support – information on 24 different available support services delivered via physical folder.	***Stress* (PSS)** – no significant differences between groups. Decrease in folder group over time from baseline to both T2 (-1.56, $p<0.05$), and T3 (-3.34, $p<0.001$)<br>*QoL* – no significant differences between groups. Improvement in folder group over time from baseline to both T2 ($p=0.0076$), and T3 ($p=0.0046$)<br><br>*Effectiveness of Intervention score = **1***	High levels of stress at baseline – particularly in folder group. Folder group also showed improvement across other measures.<br>Low adherence to intervention.
29 parents and guardians (mean age = 47.76, 86% female) of young people (mean age =	Pre-post comparison<br><br>*Timeline* – Baseline, follow up (3 months).	'Meridian' – Therapy provided through 'steps and pathways', linked to skill building and behavioural experiments.	n/a	*Stress* (PSS) – Significant reduction over time; $t(26)=3.23$, $p=0.003$, $d=0.4$.	In a feasibility study, Meridian was found to be a **safe, acceptable**, and feasible intervention.

16.83) with mental illness. [29]	*Study Quality* = Low	*Model / Development* – Consultation with young people and carers, influenced by the positive psychology framework, and supportive accountability framework. *Interactivity* – Social networking system, peer and expert moderators. *Structure* – Small 'step' modules, which participants can work through in any order. *Duration* – 3 months.		*Depression, anxiety, and stress* (DASS) – No significant difference over time. *Psychological wellbeing* (SPW) – No significant difference over time. <br>*Effectiveness of Intervention score =* ***2***	
151 carers of a person with mental illness. Intervention n = 78, control n = 73. 88.1% female. [26]	RCT <br>*Timeline* – Baseline, 10 weeks, 3 months. <br>*Study Quality* = High	Web-based mindfulness program. Basic mindfulness exercises, audio/video files, text files, private diary. <br>*Model / Development* – Mindfulness-based intervention. *Interactivity* – Audio / video files, email reminders. No reported contact with other participants. *Structure* – Recommended training; 2x10 mins/day, 6 days/week, for 8 consecutive weeks *Duration* – 10 weeks.	Wait-list control.	*QoL/burden* (CarerQoL 7-D) – Improvements in favour of the experimental group in 3/7 measures (relational problems, mental health, and problems with daily activities; effect sizes ranged from 0.30 to 0.57). *Stress* (PSS) – Decrease in perceived stress in experimental group in pre-/post-comparison and follow-up (*p*=0.002, effect size =0.66) <br>*Effectiveness of Intervention score =* ***3***	Experimental group showed significant improvements in measures of mindfulness (**FFMQ)** and self-compassion from pre-/post-intervention.
97 family members of a person with a range of mental illnesses (89% female). [27]	Pre-post comparison <br>*Timeline* – Baseline, 8 weeks, 3 months. <br>*Study Quality* = Moderate	Web-based mindfulness program. Basic mindfulness exercises, audio/video files, text files, private diary. <br>*Model / Development* – Mindfulness-based intervention. *Interactivity* – Audio / video files, email reminders. No reported contact with other participants.	n/a	*QoL/burden* (CarerQoL 7-D) – Improvements on 5/7 measures from pre-post, and 3/7 from pre to follow-up, with small effect sizes reported (all < 0.45). *Stress* (PSS) – Significant decrease in	Significant improvement in all subscales and overall score on measure of mindfulness (**FFMQ**) from pre-post, which was maintained at follow-up.

		Structure – Recommended training; 2x10 mins/day, 6 days/week, for 8 consecutive weeks<br>*Duration* – 8 weeks.		stress from pre-post (*p*=0.001, effect size =0.63), maintained at follow-up (*p*=0.013, effect size =0.73).<br><br>*Effectiveness of Intervention score* = **3**	
398 carers of people with mental or somatic illness. Intervention (n = 196, 85% female), control (n = 202), 87% female. [28]	RCT<br><br>*Timeline* – Baseline, 8 weeks, 3 months.<br><br>*Study Quality* = High	Web-based mindfulness program. Basic mindfulness exercises, audio/video files, text files, private diary.<br><br>*Model / Development* – Mindfulness-based intervention.<br>*Interactivity* – Audio / video files, email reminders. No reported contact with other participants.<br>*Structure* – Recommended training; 2x10 mins/day, 6 days/week, for 8 consecutive weeks<br>*Duration* – 8 weeks.	Wait-list control.	*QoL/burden* (CarerQoL 7-D) – No significant differences between groups.<br>*Stress* (PSS) – Significant decrease in favour of the experimental group (*p*=0.001, *d*=0.46), and over time in the experimental condition from pre- to follow-up (*p*=0.001, *d*=0.53).<br>*Burden* (Montgomery-Borgatta scale) – No between-group differences; improvements in experimental group from pre to follow-up on objective (*p*=0.001, *d*=0.26), and stress / subjective subscales (*p*=0.001, *d*=0.26).<br><br>*Effectiveness of Intervention score* = **3**	Significant improvement in mindfulness in favour of the experimental group.

Abbreviations

BPRS - Brief Psychiatric Rating Scale
BSI – Brief Symptom Inventory
CarerQoL 7-D – Carer Quality of Life Scale

CGSQ – Caregiver Strain Questionnaire
DASS – Depression Anxiety Stress Scales
EQ5D – EuroQol Group Quality of Life Measure
FFMQ - Five Facet Mindfulness Questionnaire
GHQ-28 – General Health Questionnaire; 28-item scaled version
HADS - Hospital Anxiety and Depression Scale
K10 – Kessler Psychological Distress Scale
PHQ-9 – Patient Health Questionnaire
QoL – Quality of Life
PSI – Parenting Stress Index
PSS – Perceived Stress Scale
SPW – Scale of Psychological Wellbeing
SUS – System Usability Scale
VADRS – Vanderbilt ADHD Rating Scales
ZBI – Zarit Burden Interview

Note; Primary outcome(s) denoted by **bold text**
