# Supplementary material for: Internet-Based Interventions for Carers of Individuals With Psychiatric Disorders, Neurological Disorders, or Brain Injuries: Systematic Review
Source: J Med Internet Res. 2019 Jul 9;21(7):e10876. doi: 10.2196/10876 (PMC6647754; doi:10.2196/10876)
Supplement: Multimedia Appendix 3 [file jmir_v21i7e10876_app3.pdf]

Multimedia Appendix 3: Summary of studies – carers of individuals who have survived a stroke

Participants and Study Reference	Study Design, Timeline, and Quality	Web-based intervention	Comparison / Control Group	Findings	Comments
72 primary carers of veterans who survived a stroke (95.8% female, mean age = 62.6). [31]	Pre-post comparison<br><br>*Timeline* – Baseline, follow-up within 6 weeks of intervention completion.<br><br>*Study Quality* = Low	RESCUE intervention; nurse-led telephone support and RESCUE website (consisting of fact sheets, self-management tools and resource list).<br><br>*Model / Development* – Based on relational / problem-solving model of stress (COPE).<br>*Interactivity* – weekly phone call with nurse after accessing website materials.<br>*Structure* – 4 weekly phone calls with nurse.<br>*Duration* – 4 weeks.	n/a	Depression (CES-D) – Significant reduction from pre- to post-test (p = 0.006).<br>Burden (ZBI) – Significant reduction from pre- to post-test (p = 0.005).<br><br>*Effectiveness of Intervention score = **3***	Baseline depressive symptoms and burden were both negatively associated with length of time caregiving in years.
103 carers of first-time stroke survivors. Intervention n = 51 (69% female), control n = 52 (81% female). [32]	RCT<br><br>*Timeline* – baseline, 3 months, 6 months, 9 months, 12 months.<br><br>*Study Quality* = Moderate	Caring~Web; linked websites relating to stroke and caring, educational information / tips.<br><br>*Model / Development* – Developed by researchers and web designers.<br>*Interactivity* – email forum with a nurse and rehabilitation team, facilitated email discussion with other carers.<br>*Structure* – Non-modular website<br>*Duration* – 1 year.	Care as usual.	*Depression* (CES-D) – No significant differences found between groups, or over time.<br>*Life Satisfaction (SWLS)* - No significant differences found between groups, or over time.<br><br>*Effectiveness of Intervention score = **1***	Stroke survivors whose carer participated in the web-based intervention had fewer emergency department visits and fewer hospital readmissions than those whose carer was in the control group.
38 female spousal caregivers of male stroke survivors. Intervention n = 19, control n = 19. [33]	RCT<br><br>*Timeline* – Baseline, 11 weeks, 1-month follow-up.<br><br>*Study Quality* = High	Online intervention; 5 components to provide carers with knowledge and skills, including educational videos, 'resource room', and interactive aspects detailed below.<br><br>*Model / Development* – Based on Stress Process Model, intervention refined by focus group and usability study.<br>*Interactivity* - Professional guide, online weekly chat sessions, email and message board.<br>*Structure* – Sequential weekly topics.<br>*Duration* – 11 weeks.	Information-only control – access to online 'resource room'- virtual online library.	***Depression* (CES-D)** – Carers in experimental group showed significantly lower scores at T2 and follow-up ($F_{1,29}$=6.13, p<0.01, effect size = -0.79). Additionally, higher levels of clinically meaningful change (50%+ drop) found in the intervention condition than control.	No between-conditions differences found in other caregiver measures (including mastery, self-esteem, and social support).

				*Effectiveness of Intervention score = **3***	
Older spousal caregivers of a person with dementia or stroke; n = 19, mean age = 73, 42.1% female. [34]	Pre-post comparison<br><br>*Timeline* – Baseline, 12 months.<br><br>*Study Quality* = Low	Online access to relevant information programs, and interaction with other participants.<br><br>*Model / Development* – based on the temporal model of family caring [56], and working with elderly carers.<br>*Interactivity* - Discussion forum, videophone access to contact other carers. Call centre to provide advice relating to IT usage and caring situation.<br>*Structure* – Non-modular website.<br>*Duration* – 12 months.	n/a	*Burden* (RSS) – Non-significant increase in stress over time.<br>*Mental Health* (GHQ-20) – Non-significant increase in mental health problems over time.<br><br>*Effectiveness of Intervention score = **1***	Carers reported an improvement in social support from baseline to follow-up, in addition to less need for information following the intervention.

<u>Abbreviations</u>
CES-D - Center for Epidemiologic Studies Depression Scale
GHQ-20 – General Health Questionnaire
RSS – Relative Stress Scale
SWLS – Satisfaction with Life Scale
ZBI – Zarit Burden Interview

Note; Primary outcome(s) denoted by **bold text**
