# Supplementary material for: Internet-Based Interventions for Carers of Individuals With Psychiatric Disorders, Neurological Disorders, or Brain Injuries: Systematic Review
Source: J Med Internet Res. 2019 Jul 9;21(7):e10876. doi: 10.2196/10876 (PMC6647754; doi:10.2196/10876)
Supplement: Multimedia Appendix 4 [file jmir_v21i7e10876_app4.pdf]

**Multimedia Appendix 4: Summary of studies – carers of individuals with dementia; studies with a control group**

| Participants and Study Reference | Study Design, Timeline, and Quality | Web-based intervention | Comparison / Control Group | Findings | Comments |
|---|---|---|---|---|---|
| 299 employed family carers of people with dementia (mean age = 46.9, 73% female). Intervention n = 150, control n = 149. [35] | RCT<br><br>*Timeline* – Baseline & 30 days.<br><br>*Study Quality* = Moderate | Caregivers Friend: Dealing With Dementia; text and videos modelling positive caregiving strategies. Individually tailored by questionnaire responses.<br><br>*Model / Development* - Based on the Stress and Coping model [70].<br>*Interactivity* – Video testimonials. No reported contact between participants.<br>*Structure* – Three modules, not described if delivered together or sequentially.<br>*Duration* – 30 days. | Usual care wait list control. | ***Stress*** – Greater reduction in intervention than control group over time (*p*<0.001, effect size=0.05).<br>*Depression* (CES-D) – Greater reduction in intervention than control group over time (*p*=0.009, effect size=0.02).<br>*State Anxiety* (State-Trait Anxiety Inventory subscale) - Greater reduction in intervention than control group over time (*p*=0.03, effect size=0.02).<br><br>*Effectiveness of Intervention score =* ***3*** | Mean time of using the program was only 32.2 minutes (sd = 43.5), including 59% of participants visiting once, and 41% visiting twice or more. |
| 245 family carers of people with dementia (mean age = 61.2, 69.4% female). Intervention n = 149, control n = 96). [71] | RCT<br><br>*Timeline* – Baseline, program mid-point, post-treatment (5-6 months).<br><br>*Study Quality* = High | Internet course Mastery over Dementia (MoD); Content includes problem-solving, relaxation, cognitive restructuring, & assertiveness training.<br><br>*Model / Development* – Based on meta-analysis / systematic review. Uses aspects of problem-solving, CBT etc.<br>*Interactivity* – Coach guidance and feedback.<br>*Structure* - 8 lessons & 1 booster session.<br>*Duration* – 5-6 months. | Minimal intervention; e-bulletins (every 3 weeks) with practical information on caring. No contact with coach. | ***Depression* (CES-D)** – Treatment group; significantly lower depressive symptoms post-intervention (*p*=0.034, effect size=0.29).<br>*Anxiety* (HADS) - Treatment group; significantly lower anxiety symptoms post-intervention (*p*=0.007, effect size=0.34).<br><br>*Effectiveness of Intervention score =* ***3*** | Intervention duration varied, dependent on speed at which participants completed the lessons. |
| 81 family carers of people with mild dementia. Intervention (n = 41) mean age = 67.8, 70.7% | RCT<br><br>*Timeline* – Baseline, post-treatment (8 weeks). | 'Partner in Balance' – blended care self-management. Content includes psychoeducation, behavioural modelling, and reflective assignments.<br><br>*Model / Development* – focus groups with carers of people with dementia, interviews with | Waiting list. Care as usual – nonfrequent counselling. | ***Depression* (CES-D)** – No between-group differences observed over time.<br>*Stress* (PSS) – No between-group differences observed over time. | Significant effects in favour of intervention group also observed on measures of self-efficacy and mastery. |

| | | | | | |
|---|---|---|---|---|---|
| female, control (n = 40), 60% female. [68] | *Study Quality* = High | experts, think-aloud usability tests with carers and experts.<br>*Interactivity* – Face-to-face intake and evaluation sessions with coach, email feedback from coach, forum to interact with other carers.<br>*Structure* – Carers able to choose 4 of 9 modules, with 2 weeks allocated per module.<br>*Duration* – 8 weeks. | | *Anxiety* (HADS) – No between-group differences observed over time.<br>*QoL* (ICECAP-O) – significant effect in favour of intervention group ($F_{1,60}$=4.83; p=0.032, *d*-0.58).<br><br>*Effectiveness of Intervention score =* ***2*** | |
| 49 informal carers of people with Alzheimer's disease. Intervention (n = 25) mean age = 64.2, 64% female, control (n = 24) mean age = 59, 67% female. [17] | RCT<br><br>*Timeline* – Baseline, 3 months, 6 months.<br><br>*Study Quality* = High | The Diapason program; Intervention targeted caregiver beliefs about illness/role, managing difficulties, and help/support seeking.<br><br>*Model / Development* - Based on cognitive theories of stress, literature search, and previous research [58].<br>*Interactivity* – Forum moderated by clinical psychologist.<br>*Structure* – 12 sequential, multimedia sessions.<br>*Duration* – 3 months. | Care as usual. | ***Stress* (PSS)** – No between-group differences observed over time.<br>*Burden* (ZBI) – No between-group differences observed over time.<br>*Depression* (BDI) – No between-group differences observed over time.<br>*Distress* (RMBPC) - No between-group differences observed over time.<br><br>*Effectiveness of Intervention score =* ***1*** | Qualitative findings were mixed regarding the program, with carers reporting they had high expectations, and that the program may benefit others, rather than themselves. |
| 110 carers of people with dementia (mean age = 60.9, 81% female). Intervention n = 38, attention control n = 36, control n = 36. [72] | RCT<br><br>*Timeline* – Baseline, 5 month.<br><br>*Study Quality* = Moderate | Videophone intervention, with monthly sessions, caregiver tips, educational seminars, and support group sessions.<br><br>*Model / Development* – Unclear; modelled on 'REACH II' intervention.<br>*Interactivity* – Monthly videophone sessions delivered by a certified interventionist.<br>*Structure* – 6 x 1 hour monthly videophone sessions.<br>*Duration* – 5 months. | Attention control – same amount of contact, focussed on nutrition and healthy eating. Information only control – packet of educational materials. | *Depression* (CES-D) – No between-group differences observed over time<br>*Distress* (RMBPC) – Decrease in burden in intervention condition in comparison with control condition over time ($F_{1,71}$=4.98; *p*<0.03).<br><br><br>*Effectiveness of Intervention score =* ***3*** | Carers in the intervention condition also reported comparativel y greater satisfaction with social support, and appreciation of positive aspects of caregiving. |
| 199 carers of people with Alzheimer's disease and related dementias and multiple chronic | RCT<br><br>*Timeline* – Baseline, 1 month, 3 months, 6 months. | MT4C (My Tools 4 Care); 6 module online toolkit, including 'About Me' section, FAQs, and resources. Able to add text and pictures where applicable.<br><br>*Model / Development* – Based on transitions theory [73]. | Educational control – carers given a copy of the Alzheimer's Society's 'The Progression | ***QoL* (SF-12, MCS)** – No significant differences between groups at 3 months. | Not all participants in the intervention condition accessed the programme – 73% |

| | | | | | |
|---|---|---|---|---|---|
| conditions. Intervention (n = 101) mean age = 63.4, 78% female, control (n = 98), 84% female. [62] | *Study Quality* = High | *Interactivity* – None reported.<br>*Structure* – Carers able to access each of the 6 sections as and when they choose.<br>*Duration* – 3 months. | of Alzheimer's Disease' | *Effectiveness of Intervention score* = ***1*** | accessed it at least once over 3 months. |
| 225 family carers of people with Alzheimer's disease and related dementias (mean age = 69, 75% female). [36] | RCT<br><br>*Timeline* – Baseline, 6 months, 18 months.<br><br>*Study Quality* = Low | Structural Ecosystems Therapy (SET) – structural family therapy intervention.<br>SET & Computer-Telephone Integrated System (CTIS) – SET and an information network linking carers with supportive resources.<br><br>*Model / Development* - Based on a Brief Strategic Family Therapy intervention [60].<br>*Interactivity* – Computer-telephone integrated system. Monthly 'conferences' with other carers.<br>*Structure* – Weekly (4 months), biweekly (2 months), then monthly (6 month) sessions.<br>*Duration* – 12 months. | Minimal Support Control (MSC) – short, telephone calls, consisting of active listening and empathic comments. Generic educational materials. | *Depression* (CES-D) – At 6 months; SET+CTIS showed a decrease in depression ($F_{2,124}$=3.40; $p$=0.036); SET increased, MSC relatively stable. This was maintained at 18 month follow-up.<br>*Distress* (RMBPC) – No difference between groups reported at 6 months.<br><br>*Effectiveness of Intervention score* = ***2*** | The intervention efficacy differed according to ethnicity (Cuban or white American) and 'type' of caregiver (relation to care recipient). |
| 103 family carers of people with dementia (mean age = 56.12, 84% female). Intervention n = 46, control n = 57. [74] | RCT<br><br>*Timeline* – Baseline, 3 months.<br><br>*Study Quality* = Moderate | ICare Condition (ICC); Psychoeducation, coping skills, cognitive restructuring, improving communication. Workbook with 'action plans' for carer to complete.<br><br>*Model / Development* – Adaptation of psychoeducational programme 'Coping with Caregiving' [75].<br>*Interactivity* – Embedded video clips, No reported contact between participants.<br>*Structure* – 6 sequential modules, plus 'introduction' and 'planning for the future'.<br>*Duration* – 3 months. | Education / Information-Only Condition (EOC); directed to a website containing information on dementia. | ***Stress* (PSS)** – Significant decrease over time in ICC group only; $t(45)$=3.18, $p$=0.003.<br>*Distress* (RMBPC) – No significant group differences over time.<br>*Depression* (CES-D) – No significant group differences over time.<br>*QoL* (PQoL) – No significant group differences over time.<br><br>*Effectiveness of Intervention score* = ***2*** | Non-significant trends observed in the expected direction for measures of burden, depression, and QoL. |
| 66 carers of people with neuro-degenerative disease (Alzheimer's, stroke-related dementia, Parkinson's). Mean age = | RCT<br><br>*Timeline* – Baseline, 6 months.<br><br>*Study Quality* = Moderate | Caring for Others Intervention Program<br><br>*Model / Development* - Based on psychosocial lifespan perspective.<br>*Interactivity* - Video conferencing link for a support-group intervention (1 hour/week for 10 weeks with therapist, 12 weeks without), email and forum. | Control group received no intervention. | *Depression* (CES-D) – No difference between groups at follow-up.<br>*Distress* (RMBPC) – No difference between groups at follow-up.<br>*(Mental) Health* Status (HSQ12) – No difference between groups at follow-up. | A separate, combined measure of stress (relating to their loved ones' activities of daily living, and managing behaviours) |

| | | | | | |
|---|---|---|---|---|---|
| 67.8, 76% female. [76] | | *Structure* – Weekly video conferencing for 22 weeks.<br>*Duration* – Duration of access to website not specified. | | *Effectiveness of Intervention score =* ***1*** | was computed after data collection by the researchers, and found a statistically significant effect of the intervention. |
| 72 Spanish-speaking carers of people with dementia, majority over 55 years of age. [41] | Quasi-experimental<br><br>*Timeline* – Baseline, 1 month.<br><br>*Study Quality* = Low | Website *Cuidate Cuidador* (transl. *Caregiver, take care of yourself*); Practical information, real stories from other caregivers.<br><br>*Model / Development* – Guided by team with expertise in dementias.<br>*Interactivity* - Comment section to interact with other carers, *Ask an expert* resources, videos.<br>*Structure* - 4 sessions (1 pre- and 1 post-test).<br>*Duration* – 1 month. | Control; provided with printed Spanish-language educational materials on dementia caring. | *Burden* (ZBI) – No significant difference between groups at follow-up.<br>*Depression* (CES-D) – No significant difference between groups at follow-up.<br><br>*Effectiveness of Intervention score =* ***1*** | Non-significant trends to lower depression and higher burden scores in the intervention group than control. |
| 60 carers of people with dementia (mean age = 60.69, 45% female). Intervention n = 30, control n = 30. [77] | RCT<br><br>*Timeline* – Baseline, 3 months, 6 months.<br><br>*Study Quality* = Low | ALADDIN; computerised platform, offering support and information to carers, self-monitoring questionnaires.<br><br>*Model / Development* – Not described.<br>*Interactivity* – Social networking forum with other carers, ability to request contact with clinical site.<br>*Structure* – Non-modular computerized platform.<br>*Duration* – 6 months. | Control; no contact other than 3 assessments. | *Burden* (ZBI) – No significant differences between groups over time<br>*Distress* (NPI) – No significant differences between groups over time.<br>*Depression* (BDI / Zung) – No significant differences between groups over time.<br>*QoL* (EQ-5D / QOLS) – Significant improvement in ALADDIN group at 6 months ($F_{1,16}$=5.9317; $p$=0.027).<br><br>*Effectiveness of Intervention score =* ***2*** | Although differences in depression and burden were not significant, there was a trend observed in favour of the ALADDIN intervention.<br><br>Different study sites used different measures of depression and QoL. |
| 73 informal caregivers of a person with dementia. Intervention n = 41 (mean age = 63, 61% female), | RCT<br><br>*Timeline* – Baseline, 6 months, 12 months.<br><br>*Study Quality* = High | DEMentia Digital Interactive Social Chart (DEM-DISC), helps users formulate their care and support needs, gives advice to carers dependent on their responses. Links to relevant dementia care related organisations. | Care as usual. Advised by a case manager without access to DEM-DISC. | *Quality of Life* (EQ-5D+C) – No significant difference between intervention and control group at 6 or 12 months.<br>*Distress* (NPI) - No significant difference between intervention | Carers in intervention group reported increased sense of competence at 12 months. The majority of |

| | | | | | |
|---|---|---|---|---|---|
| control n = 32 (mean age = 60.4, 50% female). [37] | | *Model / Development* – Based on recent literature and contact with people with dementia, carers, and professionals. *Interactivity* – No reported contact between participants, no reported video / audio elements. *Structure* – Non-modular website. *Duration* – 12 months. | | and control group at 6 or 12 months. *Effectiveness of Intervention score =* ***1*** | caregivers reported DEM-DISC was easy to learn, user-friendly, and were moderately satisfied with the intervention. No difference found regarding **care needs of informal caregivers** at 6 months. |

Abbreviations

**BDI** – Beck Depression Inventory
**CES-D -** Center for Epidemiologic Studies Depression Scale
**EQ-5D -** EuroQoL Five Dimentions Questionnaire
**EQ-5D+C –** EuroQoL Five Dimentions Questionnaire with Cognitive Dimension
**HADS** – Hospital Anxiety and Depression Scale
**HSQ-12** – Health Status Questionnaire 12
**ICECAP-O –** Investigating Choice Experiments for the Preferences of Older People CAPability measure for Older people
**NPI** – Neuropsychiatric Inventory
**PQoL** – Perceived Quality of Life
**PSS** – Perceived Stress Scale
**QoL** – Quality of Life
**QOLS** – Quality of Life Scale
**RMBPC** – Revised Memory and Behaviour Problems Checklist
**SF-12, MCS** – Short Form-12 item health survey, mental component summary score
**ZBI** – Zarit Burden Interview
**Zung** – Zung Depression Self Rating Scale

Note; Primary outcome(s) denoted by **bold text**
