# Supplementary material for: Internet-Based Interventions for Carers of Individuals With Psychiatric Disorders, Neurological Disorders, or Brain Injuries: Systematic Review
Source: J Med Internet Res. 2019 Jul 9;21(7):e10876. doi: 10.2196/10876 (PMC6647754; doi:10.2196/10876)
Supplement: Multimedia Appendix 5 [file jmir_v21i7e10876_app5.pdf]

Multimedia Appendix 5: Summary of studies – carers of individuals with dementia; studies without a control group

**Participants and Study Reference**	**Study Design, Timeline, and Quality**	**Web-based intervention**	**Comparison / Control Group**	**Findings**	**Comments**
6 carers of people with Alzheimer's disease (mean age = 58, 50% female). [42]	Pre-post comparison<br><br>*Timeline* – Baseline, 6 weeks, 12 weeks.<br><br>*Study Quality* = Low	Interaction with other participants in a closed Facebook support group, and posting anonymous questions to their Facebook news feed.<br><br>*Model / Development* – 'Friendsourcing' (REF – bernstien) - combining social media information seeking and crowdsourcing.<br>*Interactivity* – Messaging with other participants via closed Facebook group, messaging with Facebook friends via their news feed.<br>*Structure* – Closed Facebook group and personal news feed..<br>*Duration* – 6 weeks.	n/a	*Burden* (ZBI) – No significant difference over time.<br>*Stress* (PSS) – No significant difference over time.<br><br>*Effectiveness of Intervention score = **1***	Carer burden showed a trend towards improvement over time, but this was not significant.
28 Chinese-Canadian carers of family members with dementia (67.9% female). [78]	Pre-post comparison<br><br>*Timeline* – Baseline, 6 months.<br><br>*Study Quality* = Low	Internet-based Caregiver Support Service (ICSS); web site including caregiving information handbook.<br><br>*Model / Development* – Content developed by occupational therapists and social workers.<br>*Interactivity* – Personalised email communication with a clinician.<br>*Structure* – Non-modular website.<br>*Duration* – 6 months.	n/a	***Burden* (BSFC)** – No significant difference over time.<br>*Distress* (RMBPC) – Post-intervention score not documented in paper; reported correlated with BSFC score.<br>*Depression* (CES-D) – Post-intervention score not documented in paper; reported correlated with BSFC score.<br><br>*Effectiveness of Intervention score = **1***	Change in burden was found to differ dependent on how regularly the participant used the program, however this difference was not significant.
37 carers of people with Alzheimer's disease and related dementias and multiple chronic conditions. Intervention	Pre-post comparison<br><br>*Timeline* – Baseline, 1 month, 2 months.	MT4C (My Tools 4 Care); 4 section online toolkit, including 'About Me' section, FAQs, and resources. Able to add text and pictures where applicable.	n/a	*QoL* (SF-12, MCS) – No significant difference over time.<br><br>*Effectiveness of Intervention score = **1***	Significant increase in hope and decrease in guilt found over time.

(mean age = 63.24, 65% female). [63]	*Study Quality* = Moderate	*Model / Development* – Based on transitions theory [73].<br>*Interactivity* – Link to video 'Living with Hope'.<br>*Structure* – Carers able to access each of the 4 sections as and when they choose.			
21 carers of people with dementia (mean age = 64.44, 85.7% female). [79]	Pre-post comparison<br><br>*Timeline* – Baseline, 16 weeks.<br><br>*Study Quality* = Low	AlzOnline Positive Caregiving Classes<br><br>*Model /* Development - Curriculum developed using research into caregiver cognitive-behaviour research [67] and assertiveness training [68].<br>*Interactivity* – Message board and chatroom to communicate with other carers.<br>*Structure* – 6 interactive classes, plus links and resources.<br>*Duration* – 16 weeks.	n/a	*Burden* (CAI) – Significant decrease in the Subjective Emotion Burden subscale of the CAI from pre- to post-test (p=0.001).<br><br>*Effectiveness of Intervention score = **3***	Caregivers additionally reported a significant increase in self-efficacy, but no change regarding the positive aspects of caregiving.
22 carers of people with dementia (mean age = 66, 96% female). [38]	Pre-post comparison<br><br>*Timeline* – Baseline, 2-4 weeks post-completion.<br><br>*Study Quality* = Low	Tele-Savvy program<br>*Model / Development* – Programme draws on social cognitive and stress and coping theories.<br>*Interactivity* – Weekly group videoconferences.<br>*Structure* – 6 week structure – daily video modules (6 per week), and weekly group videoconferences.<br>*Duration* – 6 weeks.	n/a	*Burden* (ZBI) – Significant decrease from pre- to post-test (p<0.05, d=0.43).<br>*Depression* (CES-D) – Significant decrease from pre- to post-test (p<0.005, d=0.52).<br>*Anxiety* (STA-I) - Significant decrease from pre- to post-test (p<0.005, d=0.51).<br><br>*Effectiveness of Intervention score = **3***	Tele-Savvy was developed directly from the face-to-face 'Savvy Caregiver Program' (SCP). There were 8 non-completers (recruited n = 30), who displayed higher levels of burden and depression than completers.
36 family caregivers of people with dementia (72.2% female) [39]	Pre-post comparison<br><br>*Timeline* – Baseline, 9 weeks.<br><br>*Study Quality* = Moderate	Website (ADCarer.com); self-learning on caring skills, coping strategies, reducing BPSDs (behavioural and psychological symptoms of dementia) and stress.<br><br>*Model / Development* – Guided by a study investigating concerns of carers [70], CBT-based.<br>*Interactivity* – Intervention based on messaging between counsellor and carer.	n/a	***Distress* (NPI)** – Statistically significant reduction from baseline to post-test (p=0.012).<br><br>*Effectiveness of Intervention score = **3***	In care recipients, BPSD significantly reduced from baseline to post-test.

		Structure – 9 weekly topics (sequential, dependent on carers' individual needs).<br>*Duration* – 9 weeks.			
20 carers of people with Alzheimer's disease and related dementias (mean age = 63.3, 59% female). [80]	Pre-post comparison<br><br>*Timeline* – Baseline, 8 weeks, 16 weeks.<br><br>*Study Quality* = Low	STAR-C-TM intervention; telemedicine programme, focussed on identifying challenging behaviours and triggers.<br><br>*Model / Development* – Based on behaviour approaches.<br>*Interactivity* –Video-conferencing with study consultant.<br>*Structure* – weekly, 1 hour video-conferencing sessions.<br>*Duration* – 8 weeks.	n/a	*Burden* (RMBPC) – Significant decrease over time in both frequency (p = 0.03), and reaction (p = 0.003) sub-measures.<br>*Burden* (SCB) – No significant differences over time<br>*Depression* (CES-D) – No significant differences over time<br><br>*Effectiveness of Intervention score = **2***	Video-conferencing preferred to face-to-face by the majority of participants.
60 carers of people with cognitive impairment (68% dementia), mean age = 57.2. [40]	Pre-post comparison<br><br>*Timeline* – Baseline, 3 months.<br><br>*Study Quality* = High	Building Better Caregiver (BBC); interactive materials, discussions, individualised tools, and resource links.<br><br>*Model / Development* – Content developed by discussion with carers, reviewing literature, and expert opinion.<br>*Interactivity* – Bulletin boards, 'post office' to communicate with other carers.<br>*Structure* – Asynchronous workshops for 20-30 carers, new content uploaded weekly.<br>*Duration* – 6 weeks.	n/a	*Burden* (ZBI) – Significant reduction from baseline to follow-up (p<0.046, effect size=0.22).<br>*Depression* (PHQ-8; modified PHQ-9) – Significant reduction from baseline to follow-up (p<0.027, effect size=0.274).<br>*Stress* – Significant reduction from baseline to follow-up (p<0.008, effect size=0.696).<br>*Strain* (CSI) – No significant difference from baseline to follow-up.<br><br>*Effectiveness of Intervention score = **3***	Carer self-efficacy and exercise (aerobic and strength/flexibility) also improved over time.

91 carers of people with dementia, mean age = 65.51, 72% female. Chat Group n = 40, Video Group n = 51. [81]	Quasi-experimental<br><br>*Timeline* – Baseline, 6 months.<br><br>*Study Quality* = Moderate	Caring for Me (CFM) website; including dementia handbook.<br>- Chat Group Intervention; access to CFM with educational videos.<br>- Video Group Intervention; access to CFM with videoconferencing.<br><br>*Model / Development* – Lifespan perspective, expanding on stress-coping paradigm.<br>*Interactivity* – Text-based chat group vs. videoconferencing group.<br>*Structure* – non-modular website.<br>*Duration* – 6 months.	No control group – both groups received an intervention.	*Depression* (CES-D) – No significant difference between groups at follow-up.<br>*Mental Health* (HSQ 12) – Compared to Chat Group, Video Group showed significantly greater improvement ($p<0.02$).<br><br>*Effectiveness of Intervention score* = ***3***	Quasi experimental study; participants offered choice between 2 interventions. Both groups showed post-intervention improvement in self-efficacy.
Older spousal caregivers of a person with dementia or stroke; n = 19, mean age = 73, 42.1% female. [34]	Pre-post comparison<br><br>*Timeline* – Baseline, 12 months.<br><br>*Study Quality* = Low	Online access to relevant information programs, and interaction with other participants.<br><br>*Model / Development* – based on the temporal model of family caring [56], and working with elderly carers.<br>*Interactivity* - Discussion forum, videophone access to contact other carers. Call centre to provide advice relating to IT usage and caring situation.<br>*Structure* – Non-modular website.<br>*Duration* – 12 months.	n/a	*Burden* (RSS) – Non-significant increase in stress over time.<br>*Mental Health* (GHQ-20) – Non-significant increase in mental health problems over time.<br><br>*Effectiveness of Intervention score* = ***1***	Carers reported an improvement in social support from baseline to follow-up, in addition to less need for information following the intervention.

<u>Abbreviations</u>

BSFC – Burden Scale for Family Caregivers
CAI – Caregiver Appraisal Inventory
CES-D - Center for Epidemiologic Studies Depression Scale
CSI – Caregiver Strain Index
GHQ-20 – General Health Questionnaire
HSQ 12 – Health Status Questionnaire
NPI – Neuropsychiatric Inventory
PHQ-9 – Patient Health Questionnaire
PSS – Perceived Stress Scale
RMBPC – Revised Memory and Behaviour Problems Checklist
QoL – Quality of Life
RSS – Relative Stress Scale
SCB – Screen for Caregiver Burden
SF-12, MCS – Short Form-12 item health survey, mental component summary score
STA-I – State-Trait Anxiety Inventory
ZBI – Zarit Burden Interview

Note; Primary outcome(s) denoted by **bold text**
