# Supplementary material for: Internet-Based Interventions for Carers of Individuals With Psychiatric Disorders, Neurological Disorders, or Brain Injuries: Systematic Review
Source: J Med Internet Res. 2019 Jul 9;21(7):e10876. doi: 10.2196/10876 (PMC6647754; doi:10.2196/10876)
Supplement: Multimedia Appendix 6 [file jmir_v21i7e10876_app6.pdf]

Multimedia Appendix 6: Summary of studies – carers of individuals with traumatic brain injury (TBI)

**Participants and Study Reference**	**Study Design, Timeline, and Quality**	**Web-based intervention**	**Comparison / Control Group**	**Findings**	**Comments**
150 family members (40 families) of children with moderate to severe traumatic brain injury (TBI). Intervention n = 87, control n = 63. [49]	RCT<br><br>*Timeline* – Baseline, unspecified follow-up.<br><br>*Study Quality* = Moderate	Online family problem-solving (OFPS)<br><br>*Model / Development* – Includes aspects of standard problem solving and cognitive-behavioural skills.<br>*Interactivity* – 1:1 videoconferences with therapist.<br>*Structure* – 8 core sessions (consisting of a self-guided web session, and therapist videoconference) 6 additional sessions relating to specific stressors / issues.<br><br>*Duration* – Not specified.	Internet resource intervention (IRI); access to home page of brain injury resources and links. Continued any care they were receiving prior to intervention.	*Depression* (CES-D) – Family members in both groups showed greater decrease in depression if they had prior technology use (OFPS; p=0.017, IRI; p=0.035)<br>*Anxiety* (AI) - Family members in the OFPS group showed greater decrease in anxiety if they had prior technology use (p=0.006). There was a non-significant trend in this direction for those in the IRI group.<br><br>*Effectiveness of Intervention score =* ***2***	The primary aim of this study was to investigate the role of prior technology use in response to online interventions for family members.
201 carers of people with traumatic brain injury (TBI). Intervention n = 104 (86.4% female), control n = 97 (88.4% female). [43]	RCT<br><br>*Timeline* – Baseline, 10 days, 3 months.<br><br>*Study Quality* = Moderate	Brain Injury Partners; web-based intervention designed to improve family advocacy skills. Contains links and articles on brain injury, stress-reduction techniques etc.<br><br>*Model / Development* – Sources including FAST (Family Advocacy Skills Training) programme, evidence-based articles, and interviews.<br>*Interactivity* – Video examples. No reported contact between participants.<br>*Structure* – Self-directed; non-modular website.<br>*Duration* – 10 days.	Directed to the BIAUSA (Brain Injury Association of America) website. Asked to use for minimum of 30 minutes.	*Life Satisfaction* (SLS) – No change from baseline to follow-up (not assessed at post-test).<br><br>*Effectiveness of Intervention score =* ***1***	The intervention focussed specifically on improving family advocacy. Intervention group had greater improvement in knowledge, application, and intention to use skills gained.
132 carers of adolescents with TBI. Intervention n = 65 (92.3%	RCT<br><br>*Timeline* – Baseline, 6, 12, and 18 months.	CAPS (counsellor-assisted problem solving)<br><br>*Model / Development* – Based on family	Internet resources comparison; a home page with links to online	*Psychological Distress* (GSI) – Lower distress found in the CAPS group compared to IRC for participants with lower income at 6	CAPS intervention did not have an impact on carer self-efficacy.

female), control n = 67 (88% female). [44]	*Study Quality* = High	problem solving training.<br>*Interactivity* – Skype session with therapist.<br>*Structure* - 7 core modules, with up to four additional modules, plus final review module. Each module consists of an online aspect and Skype session with therapist.<br>*Duration* – 6 months.	resources. Asked to spend at least 1 hour per week accessing information.	(*p*=0.04), 12 (*p*=0.01), and 18 months (*p*=0.004).<br>*Depression* (CES-D) – No impact of group.<br><br>*Effectiveness of Intervention score = **2***	Families more vulnerable (low income) to negative post-TBI outcomes may benefit the most from a therapist-supported intervention.
37 carers of children (aged 3-9) with TBI. Intervention n = 20 (mean age = 32.8), control n = 17 (mean age = 32.5). [45]	RCT<br><br>*Timeline* – Baseline, 6 months.<br><br>*Study Quality* = High	I-InTERACT web program<br><br>*Model / Development* – Programme combines features of parent-child interaction therapy and stress management / anger control.<br>*Interactivity* – Therapist videoconference calls.<br>*Structure* – 10 core sessions, with up to 4 supplementary sessions, consisting of a self-guided web session and therapist videoconference call.<br>*Duration* – 4-6 months.	Access to study website containing links to relevant resources. Encouraged to access on weekly basis.	*Psychological Distress* (GSI) – Lower distress found in the I-InTERACT group for participants with lower income (*p*<0.01)<br>*Depression* (CES-D) – No group differences at follow-up.<br>*Stress* (PSI) – No group differences at follow-up.<br><br>*Effectiveness of Intervention score = **2***	No differences between groups on carer self-efficacy.
148 carers (117 families) of children (aged 3-9) with moderate to severe TBI. Intervention n = 41 families, 'Express' intervention n = 37 families, control n = 38 families. [46]	RCT<br><br>*Timeline* – Baseline, 6 months.<br><br>*Study Quality* = High	I-InTERACT web program<br><br>*Model / Development* – Programme combines features of parent-child interaction therapy and stress management / anger control.<br>*Interactivity* – Therapist videoconference calls.<br>*Structure* – 10 core sessions, with up to 4 supplementary sessions, consisting of a self-guided web session and therapist videoconference call.<br>*Duration* – 4-6 months.	I-InTERACT Express – abbreviated version of I-InTERACT, consisting of 7 sessions, without option for supplementary sessions.<br>———<br>Access to study website containing links to relevant resources. Encouraged to access on weekly basis for at least 1 hour.	*Psychological Distress* (GSI) – No group differences at follow-up.<br>*Depression* (CES-D) – No significant main effects of intervention at follow-up, however, regression analysis found a significant interaction for I-InTERACT and baseline depression severity (p = 0.03), with those with elevated depression in I-InTERACT showing significantly greater results than control.<br>*Stress* (PSI) – No group differences at follow-up.<br><br>*Effectiveness of Intervention score = **2***	Carers showed relatively high levels of baseline functioning, resulting in the experimenters conducting post-hoc analyses.

40 carers of children (aged 5-16) with moderate-to-severe TBI. Intervention n = 20, control n = 20. [50]	RCT<br><br>*Timeline* – Baseline, post-treatment (not time-specified).<br><br>*Study Quality* = Moderate	Family Problem Solving therapy (FPS)<br><br>*Model / Development* – Problem solving framework & TBI-specific cognitive behavioural skills.<br>*Interactivity* – Videoconference meeting with a clinician.<br>*Structure* - self-guided web materials (8 core sessions, 6 possible additional sessions), used by multiple family members, followed by clinician videoconference.<br>*Duration* – Not specified.	Internet resources comparison group; any psychosocial care they were receiving prior to the intervention. Access to a homepage of relevant links and resources.	*Psychological Distress* (SCL-90-GSI) – Lower in FPS group at follow-up (p<0.05, effect size=0.16).<br>*Depression* (CES-D) – Lower in FPS group at follow-up (p<0.05, effect size=0.16).<br>*Anxiety* (AI) – Lower in FPS group at follow-up (p<0.05, effect size=0.11).<br><br>*Effectiveness of Intervention score = **3***	The FPS group showed higher problem-solving scores than the control group, but this difference did not reach statistical significance.
9 families (12 parents) of children and adolescents (aged 11-18) with moderate-to-severe TBI. Audio group n = 5 families, non-audio group n = 4 families. [51]	RCT<br><br>*Timeline* – Baseline, post-treatment (not time-specified).<br><br>*Study Quality* = Moderate	Teen Online Problem Solving (TOPS) intervention. Families randomised to either non-audio or audio groups (same content, but with option of website read aloud).<br><br>*Model / Development* – Built off FPS intervention (see above).<br>*Interactivity* – Videoconference meeting with a clinician.<br>*Structure* - 10 core sessions, 6 possible additional sessions. Self-guided web sessions, followed by clinician videoconference.<br>*Duration* – Not specified.	No control group – both groups received intervention.	*Psychological Distress* (SCL-90-GSI) – No difference from pre- to post-treatment when families pooled. No between-group differences.<br>*Depression* (CES-D) – Significant decrease in depressive symptoms from pre- to post- when families pooled (p=0.01, d=0.80). No between-group differences.<br><br>*Effectiveness of Intervention score = **2***	Parents reported that their children displayed fewer internalising symptoms at post-intervention, but no difference in externalising symptoms.
41 family members of children and adolescents (aged 11-18) with moderate-to-severe TBI. Intervention n = 20 (mean age = 40.81),	RCT<br><br>*Timeline* – Baseline, 7-8 months.<br><br>*Study Quality* = High	Teen Online Problems Solving (TOPS) intervention.<br><br>*Model / Development* – Built off FPS intervention (see above).<br>*Interactivity* – Videoconference meeting with a clinician.	Internet resources comparison group; access to a homepage of relevant links and resources, encouraged to spend > 1 hour per	*Psychological Distress* (SCL-90-GSI) – No group differences over time.<br>*Depression* (CES-D) – Moderated by socioeconomic status (SES); lower SES in TOPS group showed improvement over time (p=0,04), higher SES in control group showed	Problem-solving improvements moderated by SES; lower income in the TOPS group showed significant improvements from baseline

control n = 21 (mean age = 41.58). [47]		*Structure* - 10 core sessions, 6 possible additional sessions. Self-guided web sessions, followed by clinician videoconference. *Duration* – 6 months.	week accessing information.	improvement over time (p=0.01).<br><br>*Effectiveness of Intervention = **2***	to post-intervention.
132 family members of children and adolescents (aged 12-17) with TBI. Intervention n = 65 (mean age = 41.91), control n = 67 (mean age = 42.84). [48]	RCT<br><br>*Timeline* – Baseline, 6 months.<br><br>*Study Quality* = High	Counselor-Assisted Problem Solving (CAPS)<br><br>*Model / Development* – Based on family problem solving framework. *Interactivity* – Skype sessions with therapist. *Structure* - 7 core sessions, supplemented by additional sessions if needed. Online self-guided module, followed by therapist Skype session. *Duration* – 6 months.	Internet resources comparison group; access to a homepage of relevant links and resources, encouraged to spend > 1 hour per week accessing information and track sites they visit.	*Psychological Distress* (SCL-90-GSI) – No significant between-group differences. Both groups showed comparable significant reduction in distress over time (p=0.007). *Depression* (CES-D) – Trend for greater improvement over time in the CAPS group vs. control. Trend becomes significant when participants completing < 4 sessions of CAPS were removed (p=0.03).<br><br>*Effectiveness of Intervention Score - **2***	Interaction between group and computer usage; in frequent users, CAPS group reported higher levels of self-efficacy than control group. Treatment efficacy not moderated by SES.

<u>Abbreviations</u>

AI – Anxiety Inventory
CES-D - Center for Epidemiologic Studies Depression Scale
GSI – Global Severity Index
SCL-90-GSI – Symptom Checklist 90 Global Severity Index
SLS – Satisfaction with Life Scale

Note; Primary outcome(s) denoted by **bold text**
